# Support–Activity Relationship in Heterogeneous Catalysis for Biomass Valorization and Fine-Chemicals Production

**DOI:** 10.3390/ma14226796

**Published:** 2021-11-11

**Authors:** Andrea Lazzarini, Roberta Colaiezzi, Francesco Gabriele, Marcello Crucianelli

**Affiliations:** Department of Physical and Chemical Sciences, University of L’Aquila, Via Vetoio (COPPITO 2), 67100 L’Aquila, Italy; roberta.colaiezzi@graduate.univaq.it (R.C.); francesco.gabriele@graduate.univaq.it (F.G.)

**Keywords:** structure–activity relationship, single-atom catalysis, green synthesis, supported metals, high-added value products

## Abstract

Heterogeneous catalysts are progressively expanding their field of application, from high-throughput reactions for traditional industrial chemistry with production volumes reaching millions of tons per year, a sector in which they are key players, to more niche applications for the production of fine chemicals. These novel applications require a progressive utilization reduction of fossil feedstocks, in favor of renewable ones. Biomasses are the most accessible source of organic precursors, having as advantage their low cost and even distribution across the globe. Unfortunately, they are intrinsically inhomogeneous in nature and their efficient exploitation requires novel catalysts. In this process, an accurate design of the active phase performing the reaction is important; nevertheless, we are often neglecting the importance of the support in guaranteeing stable performances and improving catalytic activity. This review has the goal of gathering and highlighting the cases in which the supports (either derived or not from biomass wastes) share the worth of performing the catalysis with the active phase, for those reactions involving the synthesis of fine chemicals starting from biomasses as feedstocks.

## 1. Introduction

### 1.1. Biomass Valorization: A Great Opportunity

Catalysis is the branch of chemistry that has the largest influence in global economy; in fact, it generates approximately 35% of world GDP [1]. Heterogeneous catalysis, in particular, is responsible of almost 90% of the total volume of chemical production each year [2], making it one of the most profitable industrial sectors worldwide. Such an extended economic compartment greatly affects not only the wealth level of several nations but along the decades has largely contributed to the generation of severe environmental issues, measurable not only in terms of greenhouse emissions (chemical related industry is the second largest industrial producer of greenhouse gases, after the metallurgical compartment) [3] but also in accidental/volunteer release of toxic substances into the environment. Containment actions have already been taken, since it will take years to limit the pollution level generated worldwide, such that energy production is gradually abandoning fossil sources at the advantage of more sustainable ones. Alongside the most commonly known solar, wind and hydroelectric powers, energy produced by exploiting biomasses is becoming one of the key players in the field of renewable feedstocks, since they exploit natural resources more efficiently, generating a series of useful and profitable by-products and are among the cleanest sources of energy as their production and processing generates very little polluting residues [4].

Nowadays, bioenergy covers almost 50% of all the renewable sources of energy [5]; in the EU, its employment have risen 1.94 times in the past 15 years [6] and this trend is expected to continue in the next few decades. At the industrial level, companies are more likely to employ raw materials as homogeneously as possible, in order to maximize process yields and consequently incomes: therefore, natural oils (such as canola, soy, sunflower or palm oil) are harvested with the sole purpose of being transformed into fatty acid methyl esters (FAME hereafter) to be employed as fuels, contributing significantly to the overall goal of adopting renewable and zero-emission energy sources [7]. However, despite this practice preventing the release of greenhouse gases from fossil feedstocks into the atmosphere, it is increasing land-grabbing phenomena in developing countries where these plants are effectively grown [8], tremendously endangering the local population and wildlife. The problems risen by using cultivated feedstocks as renewable sources can be somehow limited by the partial substitution of these raw materials with organic wastes from human activities, thus transforming substances that need to be disposed into precious feedstocks.

With this review paper, we are trying to build a bridge between the highly interesting topic of catalytic biomass valorization and the study of the influence that supports may exert on the total activity and selectivity of heterogeneous catalysts. In the introductory section, we will gather as high an amount of available information about biomasses discarded by human activities as possible, rationalizing and dividing them according to their origin, chemical composition and trying to provide a wide overview of their potentiality of employment in the industrial chemical sector. However, we are well aware of the huge size of this topic; therefore, we will limit our focus to the applications in the catalytic field. Quite often in modern green chemistry, biomasses are employed as catalysts or as supports, either directly (chitosan) or after a chemical transformation (activated carbons and lignin), thanks to their natural abundance of chemical species able to either directly perform directly catalyzed reactions or able to coordinate metal ions and nanoparticles. In the latter case, such functionalities might influence the reaction path, acting as directing agents towards certain products; also, the use of pure and transformed biomasses as supports will be explored in the introductory part of the paper. In Section 3, we will report those research papers that have investigated the possible influence that the structural and surface characteristics of materials have on the total catalytic activity when employed for supporting heterogeneous catalyst. As for Section 2, our focus will be on reactions involving biomass valorization, from more classical oxide-supported catalysts to MOFs, 2D materials and nanoparticles supported active phases. 

### 1.2. Biomass from Wastes to Feedstocks: Typologies, Composition, Advantages and Drawbacks

**Figure 1:**
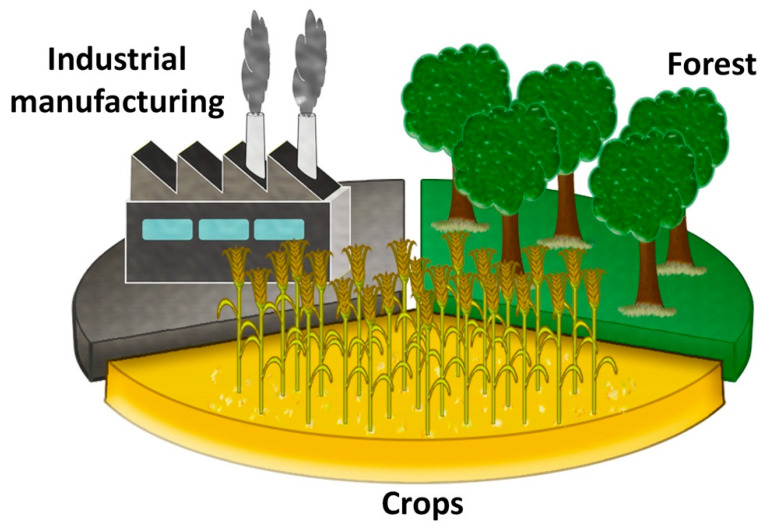
Principal vegetal biomass sources according to Tursi [11] and Kaltschmitt [12].

Biomasses of different origins, strictly intended as polysaccharidic material obtained by vegetal organisms via photosynthetic route [9], have been employed since the rise of the first agricultural societies for purposes that are diverse from their primary intended use (namely as food source for human beings or cattle): wood from forests have been used for building shelters or as fuel; the same for wastes deriving from crops, but they have also been employed as fertilizers, together with animal manure. With the development of industrial society in the 18th century, united with modern building technologies, progresses in the field of chemistry and the progressive mechanization of agricultural processes, the importance of such natural feedstocks for technological applications started to decline, touching its minimum during fossil fuels era along the 20th century. Luckily, starting from the 1970s a growing awareness among people and national governments about the environmental disaster that planet Earth was facing due to the indiscriminate use of non-renewable sources was born [10]. This pushed the society to find a way to bring back to play an enormous amount of unexploited sources that we were simply throwing away. However, the idea of using again biomasses in the same way they were employed centuries ago was definitely outdated, also for the different needs and technologies modern society has with respect to the past; moreover, other biomasses obtained by waste materials of new food industry processes (and not available only few decades ago) entered the field. According to Tursi [11] and Kaltschmitt [12], there are three main areas where biomasses from waste can be “harvested” (see Figure 1): the most important are the ones of agricultural activities and forestry residues, including residues coming from their industrial manufacturing (such as peels, shells, wood shavings and sawdust). In terms of quantity, they are closely followed by animal residues from livestock farms, sewage, algae and aquatic crops. Urban solid wastes and in general wastes originating from anthropogenic activities also fall in the biomass category, but only if they are not reusable in subsequent processing. 

As previously mentioned, the main problem with biomass wastes stands behind their heterogeneity, not only among different classes, but also within the same one; therefore, depending on the desired application, some categories are preferred with respect to others. For simple uses, such as domestic fuel, any type of biomass (properly dried in order to remove excess water) can be employed almost without any processing, thanks to their intrinsic high carbon content. This final use, which is the most primitive, varies significantly according to the wealth level of the nations: in third world nations, this type of energy accounts up to 50% of the total, against an average of 11% worldwide [10]. Nevertheless, also among the nations with the highest GDP per capita these data are not homogeneous, with the United States delivering only 3% of the country’s energy demands by using biomass, while other countries such as Finland, Sweden, and Austria are quite above the EU average (3.5%), producing 18%, 17%, and 13% of their total energy from biomass sources [13]. Despite the easiness of employment, the energetic application (together with the fertilizing use) results to be the sector with the lowest added value obtainable from these complex feedstocks. From an historical point of view, the first valuable product that was obtained by plant biomasses was paper. In the beginning of the pharmaceutical and cosmetic industrial story, many of their processes were based on molecules that can be found in various concentrations inside numerous natural matrices, mainly plant based. However, together with the progresses of the chemical industry, the tendency has been to move more and more towards molecules of synthetic origin, obtained by non-renewable more homogeneous feedstocks. Along the years, with the exponential rise of the costs of fossil materials from the 1970s [14], together with one of the wastes disposal, the concept of substituting oil-based sources with biomass wastes has become economically interesting and sustainable, even if this is not valid for any possible chemical.

**Figure 2:**
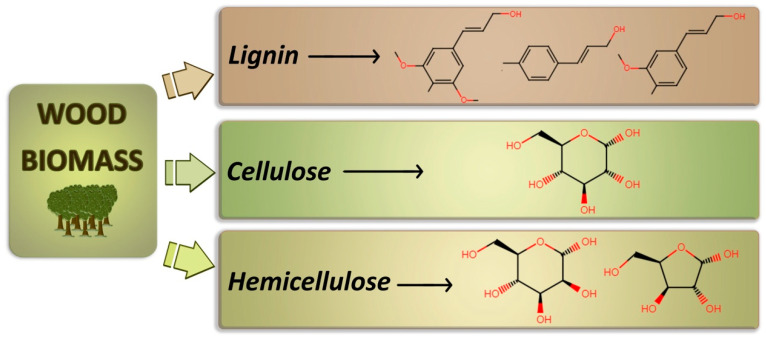
Graphical representation of the main chemical components of vegetal biomasses, i.e., cellulose, hemicellulose and lignin, with schematized molecular sub-units.

Concerning the compositional point of view, all plant-based biomasses are formed by three major constituents, namely cellulose, hemicellulose and lignin, present in different ratios depending on biomass origin (see Figure 2) [15]. Among the three, cellulose is the main component, and it is formed by extremely long linear chains of polysaccharides (D-Pyranglucose units linked by β-1,4-Glycosidic bonds [16]) arranged either in crystalline or amorphous structures, which ratio (usually between 30 and 80%) depends on the average chain length [17]. By far, cellulose is also the source exploited for the longest period of time, being the main constituent in the production of paper that is still its main application. After cellulose, hemicellulose represents the second major constituent of plant cell walls and consists of heterogeneous branched chains of polysaccharides. The amount of hemicellulose and its structure are strongly dependent on the type of vegetal source [18]. The different saccharide units are arranged with various substituents and a diverse ratio. From hemicellulose decomposition non-condensable gas, coal, and a variety of ketones, aldehydes, acids, and furans are produced [19]. Differently from cellulose, the hemicellulose chain structure consists of a mixture of sugars containing either five carbon atoms, such as xylose and arabinose, or six carbon atoms, such as glucose, galactose, mannose and rhamnose, with an average maximum molecular weight of 30,000 Da, definitely much lower with respect to the several millions of Da in cellulose chains [20]. From these constituents, it is possible to obtain several different types of molecules, including xylans, mannans, galactans, and arabinogalactans. The last constituent of vegetal biomass is lignin, contained in plant cell walls similarly to the previous ones. Its role is linking the fibers of cellulose and hemicellulose together, enhancing the resistance of the plant structure. Due to its binder role, it has to be either removed or degraded in order to extract cellulosic fibers from vegetal biomasses. Its content in the materials varies according to the type of plant and its age (from 15 to 50%); however, the elemental composition is approximately always identical, consisting of 61–65% carbon, 5–6% hydrogen and 29–34% oxygen [21]. The structure of lignin is quite far from regularity: indeed, it is a complex amorphous aromatic polymer with a three-dimensional network, with monomeric units (principally aromatic alcohols with different degrees of methoxylation [22]) bound together by oxygen bridges. Furthermore, in the lignin structure, the presence of a high number of polar groups and hydroxyl groups allows the formation of strong intermolecular and intramolecular hydrogen bonds, making it insoluble in most solvents. Being so difficultly workable and having a greater calorific value compared to lignocellulose biomass [23], only about 2% of the lignin produced in the world as waste material (300 billion tons [24]) is employed in the production of various value-added products, while the rest is employed as fuel. Therefore, there is a lot of margin for the development of innovative technologies for its valorization.

**Figure 3:**
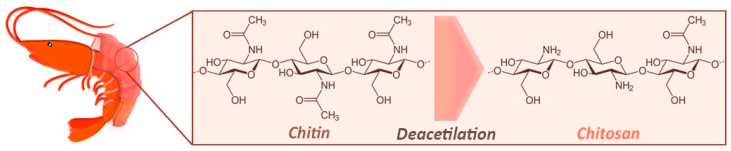
Chitosan production chain, from chitin extraction from shellfish exoskeleton to its deacetylation process to obtain chitosan.

Up to this point, we discussed prevalent biomasses coming from the vegetal world. However, the alimentary industry involves also the production of animal-based food, leaving a series of byproducts that may be exploited in the chemical industry to produce higher value products. The transformation of seafood (especially shellfish) ends up with enormous quantity of organic waste, almost entirely composed by chitin [25,26]: it is a hydrophobic polysaccharide made from acetyl-glucosamine and glucosamine grafts found in the outer skeleton of shrimps, squids, lobsters, crabs or walls of algae [27,28,29], showing many properties including good biodegradability and biocompatibility [30,31]. Chitin itself does not have enormous impact on the chemical industry, which significantly prefers to employ chitosan (see Figure 3), obtained through the deacetylation of chitin [32,33,34], which has attracted a lot attention in the green nanotechnology field. The benefits of using chitosan in technological applications include: (i) its low-cost and eco-friendly nature, (ii) its hydrophilic character, (iii) its proneness to chemical and physical modifications to customize its properties (with the insertion of coordinating groups for metal ions), and (iv) its thermal stability up to 280 °C, nontoxicity and biodegradability [28,35,36,37,38,39,40].

### 1.3. Classic Supports vs. Modern Active Supports

Once we have defined the source of feedstocks for our chemicals, it is important to go deeper into their utilization. In order to convert these materials into valuable products, the use of catalysts is often needed. It is well established that homogeneous ones can be designed to possess high selectivity towards a selected product, but the disadvantage of the non-reusability is pushing in the direction of heterogeneous counterparts, if valid alternatives are available. It is hard to find heterogeneous catalysts constituted of self-standing materials; in fact, it is preferrable to employ supported catalysts. At the dawn of the modern chemical industry, the sole role of the support was to disperse active phase, preventing its aggregation, and avoiding interferences in the reaction. Silica, alumina and carbons have been for decades as the key players in the heterogeneous supports’ world. In modern times, the shortage of feedstocks and environmental issues are forcing the production of more efficient catalysts. One way is through research focused on improving active phases. However, in some cases it is difficult (if not impossible) to improve the performances of catalysts, especially in those cases regarding noble metals; in other cases, the improvements are not environmentally or economically sustainable. For these reasons, along the years the focus has gradually moved in the direction of support development, which is usually cheaper and greener than an active species one, making the field quite attractive and with a lot of potentiality. The simplest way to modify supports properties is to act on their size and shape; oxides have been downscaled into nanoparticles, showing a great influence on the final catalytic properties, allowing a fine-tuning for preferential exposure of specific reactive sites. This can maximize the number of active sites available for reagents, improve activity, and also direct the reaction to a preferential product to achieve greater selectivity [41]. Shape could determine which specific reactive component on the oxide particle will be exposed and this, in turn, will affect the adsorption and activation of the reactants and intermediates and, therefore, on the overall catalytic properties. The synthetic approaches available so far have allowed precise control of the size of the oxide particles with significantly improved surface densities of the active sites. However, shape control, especially for particles smaller than 10 nm is still empirical. Furthermore, the presence of surface defects located at the edges and, in some cases, also at the corners of the oxide particles, resulting from shape imperfections, can contribute to the catalytic performance. As a final consideration, the majority of classic support materials for heterogeneous catalysts were developed and optimized, in the past, for hydrocarbons-based chemicals conversion; in this context, it should be noted that oxygenated compounds, such as the ones obtained by biomass-wastes, tend to be much more aggressive towards many oxidic or carbonaceous supports, as well as towards the active phases. Harsh conditions implying that the use of water, organic polar solvents, high temperatures, strong oxidants, and prolonged reaction times, may potentially degrade classical heterogeneous supports. Whenever size and shape modification are not enough, surface functionalization can be a way to imprint peculiar properties to the catalysts, either by providing the active phase a better coordination, granting its anchoring on support surface [42], or by favoring substrate adsorption, migration to the active species and products desorption [43]. In those cases, in which the desired properties are not achieved with all the possible modifications of the support, the use of materials different in nature might be necessary. Providing that not all the metals are catalytically active, there is a plethora of available, or soon to be available, supports that possibly overcome the limited number of active metals or promoters that can be deposited on the supports. For this reason, an in-depth understanding of the actual role of the support in promoting the catalytic action of active phases is of paramount importance. As already described above, biomass itself provides inhomogeneous resources, thus the adaptability of the catalysts, given also by the modulation of the supports, is already a key parameter. The currently available alternatives are numerous and can span from material having natural origins to fully synthetic ones. For both cases, we will provide an updated overview in the following sections.

## 2. Biomass for the Production of Catalytic Supports: The Cases of Activated Carbons, Lignin and Chitosan

### 2.1. Activated Carbons

Inorganic graphitic carbons are a class of highly chemically stable materials. Natural feedstocks of carbons have been mined for hundreds of years, they consist of different typologies of coal (from peat to anthracite) and are principally employed in the production of energy, even though the low-carbon transition, necessary to respond to climate change, is pushing towards an abandon of these materials. Nowadays, inorganic carbon, especially peat being the least suitable for energetic exploitation, is involved in the production of cheap and versatile supports for heterogeneous catalysts, such as activated carbons [44,45,46]; however, peat still needs shallow mining activities, united with time and energy consuming processes needed to properly dry it and make it usable for any final application. Conversely, as we reported above, biomasses are naturally rich in carbon content and are easily accessible, making them one of the simplest sources to be employed for the production of sustainable and renewable carbon-containing feedstocks, such as polysaccharides, polyols, terpenes and other compounds, the latter compounds being extremely valuable for the chemical industry and commonly present in organic wastes. The main issue in employing natural scraps as source for the aforementioned compounds is their intrinsic low concentration in such matrices, meaning the need of time and money consuming processes in order to extract and concentrate the desired compounds from the undesired matrix; sometimes instead, such compounds are directly obtainable by the decomposition of these matrices, which are though hardly degradable. However, when the heterogeneity of biomasses in chemical composition is too high, or the concentration of desired chemical species in the feedstocks is so low that the extraction process results as anti-economical, natural organic feedstocks can be reused as a green starting material for the production of activated carbons, thus avoiding the consumption of fossil sources [47]. 

Activated carbons (ACs) are well known for their high surface area value, along with their chemical and thermal stability. Depending on the origin of the feedstock, the production process might involve a pyrolysis step, not necessary for fossil carbon, but needed in the case of biomasses [48,49,50,51]. The list of starting materials employed for the production of these supports could be endless; however, the most common ones reported in the literature are summarized in Table 1. Thanks to the vastness of different alternatives well spread around the world, the exploitation of these feedstocks that are readily available, inexpensive, renewable and with distinctive chemical compositions [52], and might also aid developing countries in building up an industrial compartment and improve their life conditions.

Once the graphenic plates are obtained after pyrolysis; carbons need to be activated, meaning that they have to undergo a chemical or physical process able to transform the structure and the properties of the final materials. The most common activation methodologies are essentially two, namely: physical and chemical activation. The first one involves the treatment of the carbonized biomass at high temperatures (600–1000 °C), inert atmosphere in the presence of steam [43,53,54,55,56]; chemical activation can instead be performed directly either on carbon precursors or on the carbonized biomass that during this process are treated in the presence of chemical species at elevated temperatures [50,57]. Before the process, raw materials are initially impregnated with an acid/oxidizing agent and further dehydrating chemicals, which activate pyrolytic degradation, improve the activated carbon yield, while causing changes in the thermal precursor’s decomposition, giving rise to the formation of porous carbon materials [58,59]. This kind of activation is performed whenever the insertion of oxygenated groups (principally acids, alcohols, ethers and esters) on the surface of activated carbons is necessary [60,61]. The chemicals employed for this kind of treatment are principally strong inorganic acids, mainly H_3_PO_4_ [43,53,55,56,62,63,64,65], HNO_3_ [54] or H_2_SO_4_ [66]; however, alternative chemicals such as ZnCl_2_ [54,56], KOH [56,65] or H_2_O_2_ [65] can also be employed. Recently, alternative activation methodologies were also developed. Yang and co-workers [67] produced an activated carbon-based cobalt catalyst, starting from lignin impregnated with CoCl_2_, treated at 720 °C in presence of carbon dioxide. While high temperature is responsible for the carbonization of lignin, the CO_2_ effect on the final catalyst is the formation of small porosities (<10 nm), still generating a relatively high surface area of 400 m^2^∙g^−1^, is not present if the activation is performed in N_2_ atmosphere instead (7 m^2^∙g^−1^). The catalyst was then successfully tested for amaranth dye decomposition via oxone radical activation. Instead, Zhang and co-workers developed a brand-new method based on evaporation-induced self-assembly (EISA) to obtain hollow carbonized shells with a high surface area starting from lignin (lignin-based hollow carbon—LHC) [68]. Briefly, lignin was deposited from a solution onto MgO nanoparticles, followed by carbonization in inert atmosphere at 600 °C; afterwards, the template was removed using HCl as etching agent. In the end, they deposited ZnO nanoparticles onto the support and tested for photocatalytic hydrogen evolution reaction.

**Figure 4:**
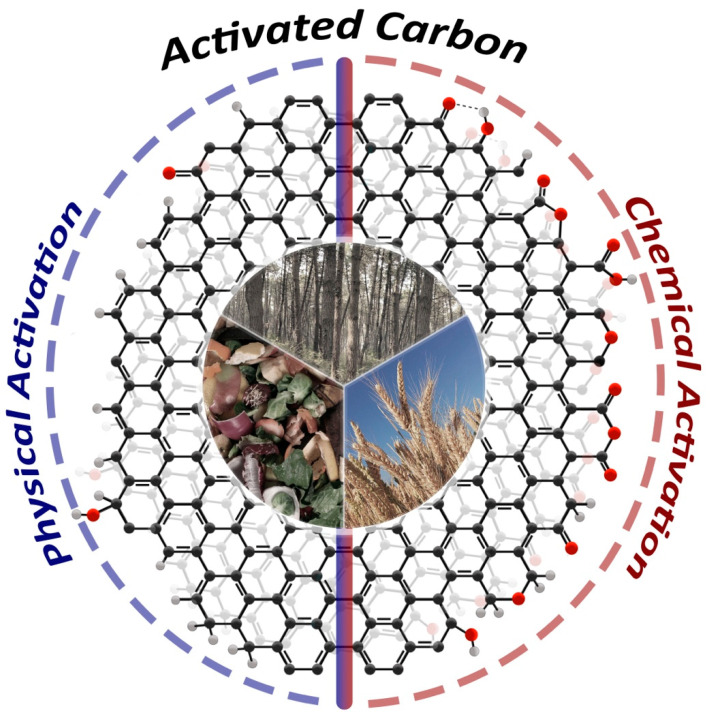
Center: activated carbons principal biomass precursors, namely forest scraps, agricultural discards, and industrial processes wastes (mainly from food and paper industries). External border: schematic representation of graphene-like plains obtained after biomasses pyrolysis; on the left side is depicted the typical average chemical speciation of graphitic domain borders in the case of physical activation (mainly H-terminations with few oxygenated ones); on the right side is reported the typical average chemical speciation of graphitic domain borders in the case of chemical activation (various oxygen-containing functional groups).

The tunability of the synthetic procedures, by changing the type of activation but also the nature of the support [69], gives these kinds of supports several degrees of freedom regarding the desired structure and chemical composition of the surface (Figure 4), in order to exploit the final catalysts at their best, once the active phase is deposited. Unfortunately, sometimes the activation procedure does not leave a material with the desired properties. For this purpose, post-synthetic modifications are used. Usually, these procedures are employed to introduce a higher amount of oxygenated functional groups and involve the further use of oxidizing agents, such as HNO_3_ [66,70], H_2_O_2_ [66] or (NH_4_)_2_S_2_O_8_ [66,71]. Much more seldom are the cases when the activation procedure leaves the surface of the activated carbons over-oxidized for the desired application and a restoration of the surface regularity, typical of graphene platelet borders, is necessary. The simplest way to do obtain such materials is a simple repetition of the pyrolysis step in inert atmosphere, at a different time and temperature of treatment [72].

The final scope of producing customized activated carbons is to provide the best support for catalyst production for a selected chemical reaction. This means depositing the active phase on a support, able not only to stabilize the active phase, but if possible being directly involved in promoting the reaction. Dealing with traditional nanoparticles active phase, the papers from Lazzarini and co-workers deeply investigated the relationship existing between the amount of oxygenated functional groups present on the surface of the supports and the best type of reaction that can be performed keeping unaltered the deposited active phase [43,70]. They found that physical activation does not introduce a large number of oxygenated groups and better maintains the graphene-like regular structure, improving activity and selectivity towards hydrogenation reactions, as tested for transfer hydrogenation of resorcinol to 1,3-cyclohexanedione [43] and hydrogenation of 1-methyl-1-cyclohexene to methyl cyclohexane [70]. Conversely, chemical activation is responsible for the insertion of oxygenated groups that break the regularity of the conjugated structure, promoting debenzylation reactions. Rusanen and co-workers studied the effect that different activations of carbons have on the conversion of xylose into furfural, also bringing into play biomass valorization [54]. They compared Fe-based catalysts prepared using carbons activated either with steam, ZnCl_2_ or HNO_3_, or with homogeneous FeCl_3_. The best results were obtained with steam activated supports that showed the highest number of hydroxyl groups, introducing labile acid protons thus promoting furfural yield. Techikawara et al. found that weakly acid activated carbons stabilize extremely small Ru amorphous nanoparticles having a thin oxide layer. The catalyst was employed to convert chitin building blocks, i.e., *N*-Acetylglucosamine (NAG) into acetylglycine (AcGly), with *N*-acetylmonoethanolamine (AMEA) as reaction intermediate [73]. Finally, Quayson and co-workers used activated carbons to support an unusual active phase: they successfully grew fungi of the *Aspergillus oryzæ* species (specifically modified to express *Fusarium heterosporum* lipase) [74], exploiting the mineral residues of the carbonization process. Such microorganisms were employed for the production of fatty acid methyl esters (FAME) from vegetal oil [75].

### 2.2. Lignin Nanocapsules

Some of the biomasses that are utilized in the production of activated carbons (either vegetal or animal) can also work as catalysts supports without undergoing the pyrolysis and activation processes, even if the cases in which they are employed in this manner are quite limited. Vegetal wastes being the main source for carbonized supports, we will first report some examples with the use of lignin for such applications that started to be employed for catalytic applications only in the last few years [84]. The research group of Prof. Saladino used lignin obtained via organosolv process as byproduct from the paper industry as supports for enzymes in oxidation reactions. In 2018, they reported tyrosinase immobilization via direct adsorption, encapsulation or layer-by-layer deposition, with or without the use of glutaraldehyde as reticulating agent, onto lignin nanoparticles supports, which were found to be stable to tyrosinase activity. After enzyme loading, they showed catalytic activity in the synthesis of catechol derivatives, with the lowest conversions in the case of encapsulated enzyme and the highest in the case of glutaraldehyde-assisted co-immobilization of tyrosinase in the presence of bovine serum albumin [85]. Similarly, in 2019, they reported the same synthetic procedures for laccase immobilization on lignin NPs. The new catalysts converted alcohols to aldehydes with high selectivity when 2,2,6,6-tetramethylpiperidin-1-yloxyl (TEMPO) was used as a redox mediator [86]. Instead, Yang and co-workers proposed the use of ß-cyclodextrin attached to lignin using epichlorohydrin as cross-linking agent. The catalyst was then employed in an oxidation reaction of benzyl alcohol to obtain benzaldehyde; the catalyst showed good selectivity (>99% in the best reaction conditions) using H_2_O_2_ as the oxidant. The weak interactions between the active phase and lignin support efficiently favored oxidation reaction of benzyl alcohol. The catalysts also demonstrated good recyclability, with no appreciable loss of catalytic activity, making it suitable to be used for green synthesis of benzaldehyde [87]. Chen and collaborators found a different way to use lignin as catalyst support, namely by forming a lignin-phenolic resin employed as support for palladium nanoparticles. The incorporation of lignin in the phenolic resin resulted in a significant size decrease in the supports united with an increment of the surface roughness and consequently of the specific surface area of the resulting material, thus augmenting the active phase loading. The final catalyst showed excellent reducing activity towards harmful metal ions (Cr^VI^) and toxic dyes [88].

### 2.3. Chitosan

Apart from lignin, chitosan is the second largest key player in the world of biomass wastes, and thanks to its abundance coupled with the ease of functionalization, it has become largely employed as catalyst support. Before being employed for technological applications, chitosan necessitates a crosslinking process (either covalent or ionic), in order to decrease porosity and swelling, but affecting the accessibility of metal ions; however, this process strongly improves thermal stability and mechanical properties [89]. The obtained material has increased robustness, minimizing dissolution phenomena. Indeed, cross-linking increases stability, mechanical strength, and chemical resistance [90]. Post-synthetic functionalization can introduce chelating chemical species on the same functional groups exploited in the crosslinking process: among the various possibilities, C-N bonds are extremely efficient in the chelation of metal ions. The most exploited is the imine formation, able to bear almost any metallic cation, that can either come from wastewater (in the case of bioremediation applications) or synthetically coordinated in the case of catalyst production. Concerning the latter case, Pd catalysts have been employed for decades in coupling reactions, supported onto various oxides [91] or activated carbons [92]. However, the Schiff-base coordinated Pd species inserted inside the chitosan network displays excellent catalytic behavior. Baran and co-workers produced imine between chitosan -NH_2_ groups and (E)-4-(2-oxoethylideneamino)benzoic acid [93,94], 2,2’-Pyridil [95], or (E)-2-((4-isopropylphenyl)imino)-1,2-di(pyridin-2-yl)ethanone (NSB) [96], able to graft Pd^II^ cations and successfully performed Suzuki cross-coupling of phenyl boronic acid and aril halides. Similar catalysts were obtained by Hardy and collaborators with chitosan functionalization via imine formation with 2-pyridinecarboxaldehyde and the subsequent coordination of Pd acetate species [27]. Schiff-bases have been also employed to bear metals different from Pd. Thatte and co-workers linked 2-(2-aminoethyliminomethyl)phenol via the S_N_2 reaction to cyanuric chloride-functionalized chitosan; the obtained groups were employed to carry Mn, Cu or Co ions and tested for liquid phase oxidation reaction [97]. Chitosan crosslinking is not performed exclusively in intramolecular way, various examples of bridging onto fibrous supports are also present in the specialized literature. The team of Alshehri and co-workers synthesized chitosan-MWCNTs, connected with glutaraldehyde bridges; the composite was then decorated with silver nanoparticles. Adding chitosan to MWCNTs improved their water solubility and nanoparticles stability. The obtained catalyst was used in the aqueous catalytic reduction of 4-nitrophenol [98]. A similar material, obtained simply exploiting combined entanglement and salifying effects of chitosan chains with poly(methacrylic acid), was prepared by Shao and collaborators [99]. A fiber prepared by electrospinning of this composite was employed as a support for palladium nanoparticles and tested in the Mizoroki–Heck cross-coupling reaction. The same reaction was tested also with 2-pyridinecarboxaldehyde functionalized composite support bearing Pd, Co or Ni. Being constituted by a huge amount of hydroxyl and amino groups, chitosan does not always need to be functionalized to be able to carry catalytically active chemical species. Liew and collaborators deposited chitosan onto magnetic nanoparticles, stabilizing the polysaccharide with phosphate bridges. The obtained support was used to deposit ruthenium nanoparticles and used for nitroarenes reduction [100]. The team of Mahdavinia and collaborators embedded magnetic laponite inside chitosan fibers, stabilized with phosphate bridges. After the loading of Cu^2+^ cations, the catalyst was successfully tested in the cycloaddition of alkyl halides and terminal alkynes with sodium azide [101]. Finally, Safaiee and co-workers coordinated vanadyl species directly using hydroxyls and amino groups present along chitosan chains. The obtained material was employed in the synthesis of 1,4-dihydropyridines and 2,4,6-triarylpyridines via anomeric based oxidation [102].

## 3. Support Materials Directly Influencing Reactions for Biomass Conversion into Fine Chemicals

### 3.1. Oxide Supports

**Figure 5:**
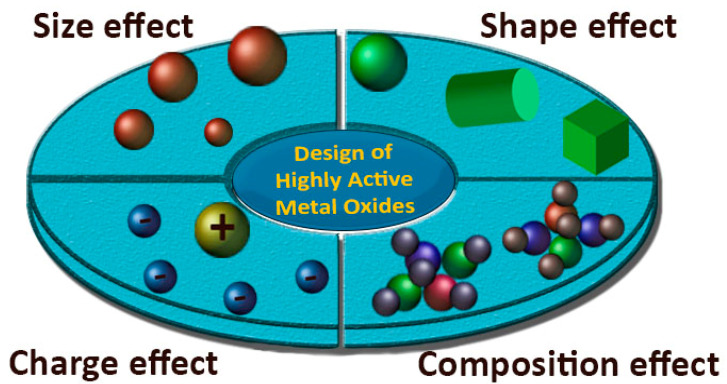
Schematic illustration of metal oxides (MOs) fine-tuning through modification of their size, shape, composition and electronic/chemical state.

The preparation of catalytically active materials possessing the proper activity, selectivity, stability, and economic sustainability, through the fine customization of metal oxides (MOs), might be considered the best goal in the field of catalysts manufacturing. Size, shape, porosity, redox and electronic modifications via specific synthetic and post-synthetic methods can provide a way to deeply modify not only the properties of the active sites themselves, but the metal–support interface activity too, obtaining highly active catalysts, with enhanced stability, for actual energetic and environmental applications (Figure 5).

Within the last few decades, several types of catalysts, which in general can be divided into noble metal (NMs)-based catalysts and NMs-free metal oxides (MOs), such as single metal oxides, mixed metal oxides (MMOs), zeolites, hexa-aluminates, perovskites, hydrotalcites and spinels, have been employed, alongside those for energy and environmental applications, also in the strategic field of biomass valorization [103]. However, the scarcity and extremely high cost of (NMs)-based catalysts, render compulsory the development of highly active, stable and selective catalysts that, nevertheless, should be affordable. Conversely, MOs synthesized starting from earth-abundant and cheap transition metals are attracting significant attention as alternatives to rare and expensive noble metals, due to their peculiar properties, such as enhanced redox properties, thermal stability and catalytic performance in combination to their lower cost. A series of the latter MOs will be briefly reviewed in their most relevant properties.

#### 3.1.1. Titania

**Figure 6:**
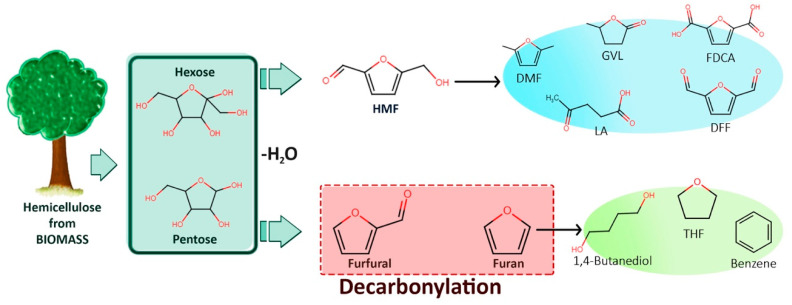
Schematic representation of the main chemical products derived from hemicellulose.

As the global economy gradually shifts from non-renewable sources to renewable ones, it is essential to develop technologies able to convert platform molecules from sustainable sources to industrially relevant products. The conversion of biomasses and their derivatives to pivotal chemicals has become a central target of academic research [104,105,106]. In recent years, researchers have developed several routes to obtain high surface area nanostructured titanium dioxide. These nanostructures were used for several applications as supports, among others, for the fabrication of several electrochemical energy conversion and storage devices [107] or for selective catalytic processes toward chemical production from biomass sources [108]. Among biomass sources, furfural is a fundamental chemical obtained from ligneous feedstocks and serves as a versatile model compound for the synthesis multifunctional molecules (Figure 6).

Having in mind a deep investigation on the metal–support interactions, a recent study showed how the single-atom site Ir catalyst, loaded onto defective metastable phase of TiO_2_, are able to exhibit excellent catalytic performance in the hydrogenation of furfural (FAL) to furfuryl alcohol, displaying excellent conversion (99%), high selectivity (99%), and good stability, even superior to the single-atom site Ir species supported onto mesoporous graphitic carbon nitride, as well as Ir nanoparticles [109]. By means of first-principle simulations, along with experimental techniques, authors revealed that the excellent catalytic performance can be attributed to the specific strength of interactions betwixt the active metallic sites (with different geometric and electronic features) and the reactant molecules, due to the tuning of the defective supports. In a recent paper, the decarbonylation of FAL to furan was examined by means of highly dispersed Pd nanoparticles (NPs) immobilized on various titania supports [110]. In particular, sodium titanate nanotubes (TNTs) with a high surface area behaved as a functional support for the creation of highly active small Pd NPs, exhibiting superior catalytic performance for the liquid-phase decarbonylation reaction of FAL without the use of additives, in comparison with the traditional supported Pd catalysts. An evident relationship was observed between reaction rates and particle size, and further analyses suggested that zero-valent small Pd NPs possessing an increased fraction of coordinatively-unsaturated Pd atoms located at the edge/corner sites are the highly active species for this specific reaction. As a matter of fact, valorization of lignocellulosic biomass is expected to strongly contribute to the realization of a sustainable and carbon–neutral society, that is finding attractive and sustainable alternatives to conventional chemical syntheses from fossil resources.

Starting from Japanese cedar biomass and using Pt-based catalysts supported over different systems such as active carbon, alumina, zirconia, and titania, the effect of the catalyst support on the aromatic monomer production yield was studied, finding that the titania-supported Pt catalyst (5% Pt/TiO_2_) afforded the greatest yield (36.2%), working for 1 h at 673 K [111]. Such a catalyst confirmed a good yield (ca. 25%), even if with different product selectivity, until the fourth recycle; however, a slight aggregation of Pt particles, thus justifying a partial deactivation of the catalyst with a contemporary partial TiO_2_ reduction and coke formation, was observed. Within the field of biomass valorization, reductive catalytic fractionation (RCF) has emerged as a promising method to extract and selectively depolymerize lignin into more valuable monomers and dimers from raw biomass feedstock, even working at or near atmospheric pressure and without the use of hydrogen [112]. Furthermore, this procedure is commonly performed in the presence of protic solvents in a temperature range between 453 and 523 K, using supported transition metal catalysts (e.g., ruthenium, palladium, or nickel) with H_2_ or alternative hydrogen sources (e.g., methanol, isopropyl alcohol, or formic acid) as reducing agents. Owing to the enhancement of the possibility of a direct use of these lignin-derived monomers as aromatic fuels or as fuel additives, their volatility should be increased by decreasing their high oxygen content (20–25 wt%), resulting in high boiling points, through hydrodeoxygenation (HDO) procedures, which have proved to be an effective reduction method for aromatic C–O bonds cleavage via hydrogenolysis. The capability of titania-supported bifunctional molybdenum-based polyoxometalates (POM/TiO_2_) in promoting tandem HDO and alkylation reactions, for the conversion of lignin-derived oxygenated aromatics into alkylated benzenes and alkylated phenols with high yields, was recently described [113]. This gas-phase study was performed on anisole and 4-propylguaiacol, as model compounds, using a packed-bed flow reactor.

**Scheme 1:**
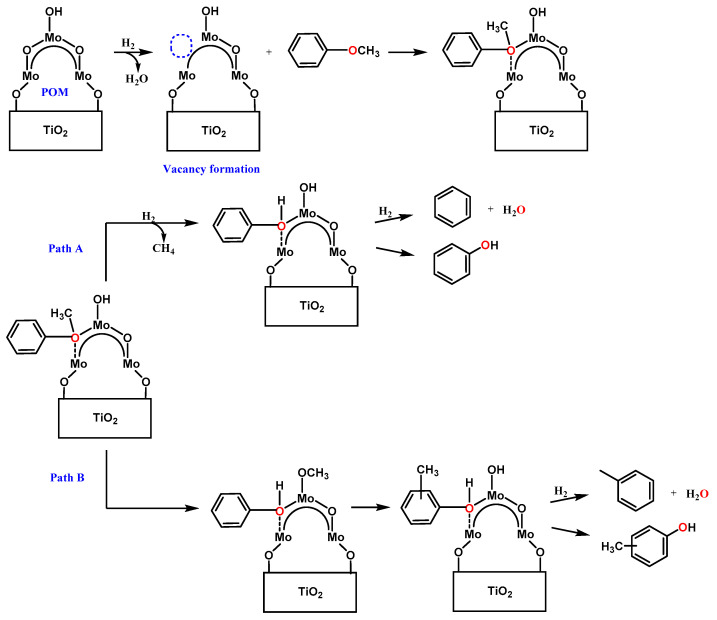
Proposed reaction network (a reverse Mars van Krevelen reaction pathway) for coupled HDO and alkylation reaction on the surface of POM/TiO_2_.

**Scheme 2:**
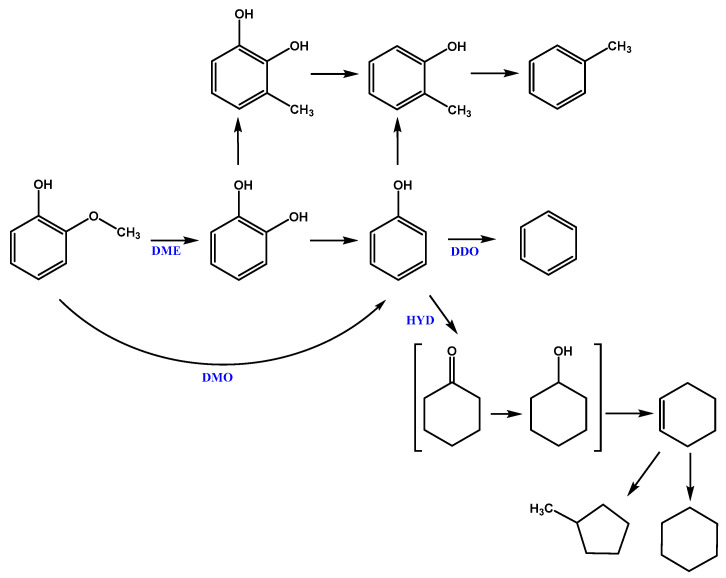
Reaction pathway for guaiacol conversion (demethylation (DME), with the cleavage of Csp^3^-OC_Ar_ bond to produce catechol (o-hydroxyphenol), followed by direct deoxygenation (DDO) to form phenol, and demethoxylation (DMO) in which the Csp^2^-OCH_3_ bond is broken, yielding phenol directly).

Both characterization and reactivity studies revealed that Lewis acid sites act cooperatively with neighboring Brønsted ones, simultaneously promoting alkylation and HDO activity. The proposed reaction mechanism involves the ether bond activation on a Lewis acid site; afterwards, the catalyst vacancy formation induced by hydrogen (path A, Scheme 1) is followed by methyl transfer and/or C-alkylation (Path B, Scheme 1). Reasonably, the balance optimization of these two functionalities (i.e., the Brønsted and Lewis acidity) with modulation by metal substitutions, will further improve the HDO/alkylation selectivity of the catalyst. With the aim of finding an efficient and selective heterogeneous catalytic system for the HDO of phenolic compounds, several TiO_2_, ZrO_2_ and TiO_2_-ZrO_2_ mixed oxides supported NiW catalysts were recently studied in the HDO of guaiacol (ortho-hydroxyanisole, Scheme 2) [114]. The three tested supports, i.e., titania, zirconia and titania-zirconia mixed oxide (TZ(80/20)), besides the presence of Brønsted acid sites, they also exhibited more active basic sites (zirconia).

Generally speaking, the supports are themselves able to catalyze the guaiacol HDO obtaining catechol, phenol and a considerable number of methylated compounds. In addition, the supported NiW systems are able to, to a remarkable degree, affect the product distribution towards completely deoxygenated compounds, in comparison with the supports alone. The ratio between titania and zirconia in the mixed oxides system was changed in order to evaluate the effect of TiO_2_ content on the catalyst activity. Multifunctional catalysts are more suitable in this kind of reactions, due to a combination of Brønsted acid sites, acid strength, and surface vacancies close to the active sites that are necessary to promote the direct elimination of the methoxy groups via hydrogenolysis of the aromatic C–O bond. Specifically, Brønsted sites may be responsible for the deoxygenation/dehydration steps; on the other hand, the surface vacancies at the metal sites (either oxygen or sulfur vacancies) are capable to promote the direct deoxygenation (DDO). Accordingly, high acidity values seemed to favor the demethylation (DME) route, obtaining catechol as the main product; differently, a medium acid strength appeared to behave as the active species for the cleavage of the aromatic C–OCH_3_ bond. The catalyst supported on TiO_2_ was the most active per atom of metal amount, i.e., 62% higher HDO activity with respect to the NiW/TiO_2_-ZrO_2_(80/20) catalyst. The titania contained in the support was a determinant to obtain completely deoxygenated compounds, such as cyclohexane and benzene (Scheme 2).

#### 3.1.2. Alumina, Silica and Molecular Sieves

MCM-41 is one of the most common mesoporous molecular sieves and is characterized by well-ordered channels with controllable uniform pore size of 2–10 nm, as well as a large surface area value; these two factors allow a molecular fast diffusion (even large organic molecules) with respect to microporous molecular sieves. This type of material allows a facile doping with several heteroatoms, substituting silicon into MCM-41 framework, thus assigning to the final material specific and modular catalytic properties, as in the case of V-substituted MCM-41 (V-MCM-41), which can be prepared using diverse synthetic procedures [115,116]. The main goal is to gain high stability of the metal-containing mesoporous materials, in order to avoid the leaching of isomorphously substituted metal species in the silicate framework under reaction conditions, therefore, favoring the formation of aggregates that might clog MCM-41 pores. V-MCM-41 showed good activity in several liquid phase oxidation reactions using H_2_O_2_ as oxidant. The activity and selectivity of these catalysts depend on oxidation state, coordination condition, dispersion and stability of the vanadium species contained in the siliceous matrix.

**Scheme 3:**
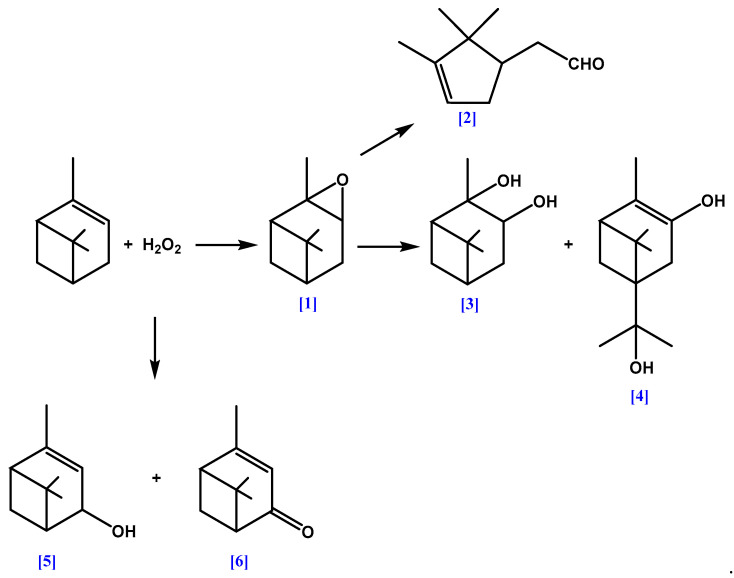
Products obtained from α-pinene oxidation: α-pinene oxide (**1**), campholenic aldehyde (**2**), 1,2-pinanediol (**3**), trans-sobrerol (**4**), verbenol (**5**) and verbenone (**6**).

In a recent paper, the catalytic activity of a series of V-M(x) (vanadium-containing molecular sieves) compounds was tested for the oxidation of α-pinene with H_2_O_2_, at 70 °C [117]. The main observed reaction products were verbenone, trans-sobrerol and campholenic aldehyde (Scheme 3). The α-pinene conversion and the distribution of products is depending on the V content in V-M(x) materials. UV–Vis-diffuse reflectance spectra confirmed the presence of two different types of V^δ+^ species. Authors suggested that V may be incorporated in two different sites of the framework, namely inside the walls constituting the MCM-41 structure, or at the wall surface. Furthermore, oligonuclear (V^δ+^···O^δ−^···V^δ+^)_n_ nano-clusters were identified, coming from an incipient oligomerization of V species. The catalysts’ higher turnover numbers (TONs), coupled with a lower metal content, testifies to the high dispersion and catalytic efficiency of the isolated V sites present at the wall surface of MCM-41 channels. Contrarily, it was proved that a higher V content negatively affects the overall catalytic performances of the material, due to the high spillover effect: spillovered V occupies extra-framework sites, which are responsible for the consumption of peroxide and water decomposition, resulting in lower H_2_O_2_ efficiency and substrate conversion. The acid–based properties of V-M(x) influenced the distribution of products formed via α-pinene oxide isomerization, taking place over Lewis acid sites, to campholenic aldehyde, while Brønsted ones are responsible of the formation of 1,2-pinanediol and transsobrerol through hydrolysis and opening of oxirane ring. 

**Scheme 4:**
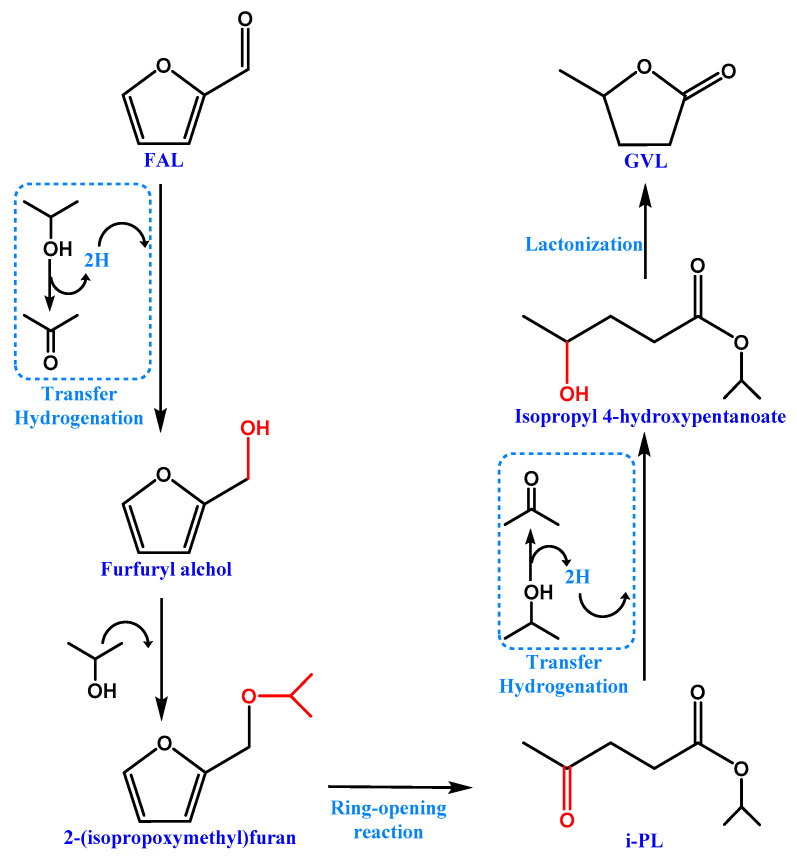
Pathway for the furfural (FAL) to γ-valerolactone (GVL) overall transformation.

As already stated, development of biomass-derived chemicals is of large interest to many researchers, among which either 5-hydroxymethylfurfural (HMF) or furfural (FAL) are considered to be important bridging molecules between biomass and specialty chemicals. HMF selective oxidation to obtain 2,5-diformylfuran (DFF) has received particular attention because DFF is considered a valuable reaction intermediate for pharmaceuticals, fungicides, heterocyclic ligands, and furan-based biopolymers manufacturing processes [118,119]. At the same time, FAL remains a versatile biomass-based pivotal molecule obtained from agricultural waste, such as corn stover and wood chips, and used as feedstock to produce isopropyl levulinate (i-PL) and γ-valerolactone (GVL) by a multistep series reactions, including hydrolysis, ring-opening, Meerwein−Ponndorf−Verley (MPV), and lactonization reactions (Scheme 4) [120,121].

In 5-hydroxymethylfurfural (HMF) heterogeneous oxidation to 2,5-diformylfuran (DFF) (Figure 6) using molecular oxygen under ambient pressure, the catalyst based on vanadium (V) oxide supported on copper mordenite zeolites (Cu−MOR), showed elevated DFF yield (91.5%), united with good reusability [122]. Additionally, alsoworking under ambient pressure, DFF yield was maintained up to 72.1%. Structure−activity relationship analyses evidenced a strong interaction betwixt the Cu species present in the zeolite framework and the guest V sites responsible for the remarkable performance. As stated by the authors, due to this strong interaction with the framework Cu species, the superficial V species of V_2_O_5_@Cu−MOR may principally be in the tetrahedral configuration, with vanadium in a 5+ oxidation state, thus improving the mobility and reducibility of the lattice O species, which ultimately may enhance the activity in the reaction. Such host−guest interaction, improved either the activity or the stability of V surface species, allowing the reusability of the heterogeneous catalyst. In another paper, bifunctional heterogeneous catalysts (HZ-ZrP) prepared by using H-ZSM-5 zeolite as support for zirconium phosphate (ZrP) was successfully employed for a one-pot conversion of biomass-derived FAL to high added value products (Scheme 4) [123].

By adjusting the loading of ZrP, Lewis-to-Brønsted acidity ratio may be modified, along with the acid strength of the catalysts. According to the literature, Lewis acid sites based on the Zr^4+^-O^2−^ pair in the ZrP active component, greatly affecting the transfer hydrogenation step; in contrast, Brønsted acid sites of zeolite play an important role in both the lactonization reaction of alkyl 4-hydroxypentanoate and the ring-opening reaction of furfuryl ether (Scheme 4). Under optimized conditions (453 K for 10 h), with isopropanol as hydrogen donor, a 93.8% total yield of i-PL and GVL, was obtained.

**Scheme 5:**
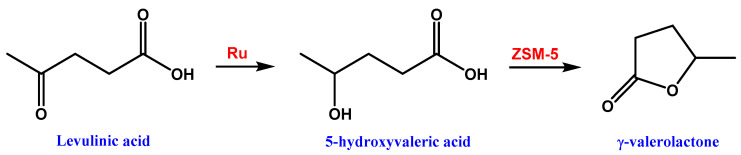
Reaction pathway of levulinic acid (LA) hydrogenation to γ-valerolactone (GVL) over Ru/ZSM-5 catalysts.

Authors observed that the catalytic activity diminished during recycling experiments due to coke formation (which covered the active sites and blocked the pores), even if the catalyst may be restored, after calcination. In another approach, GVL has been obtained starting from levulinic acid (LA), i.e., one of the most important and versatile platform molecules derived from low-cost lignocellulose wastes, by means of initial hydrogenation and then dehydration–lactonization steps (Scheme 5), over ruthenium catalysts supported on various ZSM-5 zeolites, having different structure features [124].

In this paper, authors deeply investigated the influences of types of Al species (i.e., in-framework tetrahedral aluminum (Al^IV^) and extra-framework octahedral aluminum (Al^VI^) peaks, detected by ^27^Al CP-MAS NMR), structure of support, Ru size and eventual leaching, and coke deposition, on the activity and stability of the heterogeneous catalysts. It was confirmed that the tetrahedral-coordinated Al in ZSM-5 framework contributed to the favorable ruthenium-support interaction, and thus suppressed the aggregation and leaching of ruthenium, under the acidic environment, during recycling. Additionally, the suitable pore volume and channel network connectivity successfully prevent the deposition of carbonaceous residues on the surface and channels of ZSM-5. The most active catalysts showed to be recyclable at least ten times keeping its activity intact (conv. > 95%, yield > 85%).

As known, biomass-derived glycerol is obtained as a biodiesel manufacture byproduct, coming from vegetable oils transesterification [125] and/or through hydrogenation/hydrogenolysis of simple carbohydrates and sugar alcohols, directly derived from abundant renewable lignocellulosic biomass. It follows that glycerol is ranked among the top 12 biomass-derived building blocks in the biorefinery field [126]. Among others, an interesting glycerol-based rection is its aqueous-phase hydrogenolysis to obtain 1,3-propanediol (1,3-PDO). This product can be employed in the production of polytrimethylene terephthalate (PTT), hence representing one of the most interesting compounds obtainable from glycerol. Sadly, the formation of the vicinal diol, i.e., 1,2-propanediol (1,2-PDO), is frequently favored, with a consequent 1,3-PDO/1,2-PDO low ratios that are commonly obtained.

**Figure 7:**
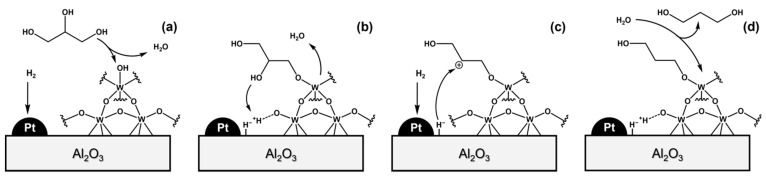
Reaction mechanism proposed for glycerol hydrogenolysis to 1,3-propanediol (1,3-PDO) over a Pt/WO_x_ supported on alumina. In brief: (**a**) glycerol coordination on WO_x_ via first water molecule elimination and H_2_ heterolitic splitting over Pt NPs; (**b**) second water molecule elimination via formation of carbocation; (**c**) carbocation reduction through the interaciton with spillovered H^−^, beared onto Al_2_O_3_ support; (**d**) 1,3-PDO desorption and W–OH active site restoration through water splitting.

To shed light on this point, three aluminum oxide-based materials and a H-ZSM-5 zeolite supports were used to deposit bimetallic Pt-WO_x_ catalysts to unravel the structure–activity relationships for the glycerol hydrogenolysis reaction towards 1,3-PDO (Figure 7) [127]. In order to enhance the selectivity (1,3- vs. 1,2-PDO), the surface density of tungsten species and the intimate contact between Pt and WO_x_, were found as the key parameters. Indeed, surface W density modulates the formation of polytungstates, which are the sole chemical species able to produce the weak Brønsted acidity required to selectively produce 1,3-PDO. From a comparison between the H-ZSM-5 and the alumina supports, authors highlighted that a slight increment of Brønsted acidity strength (using H-ZSM-5) was detrimental for 1,3-PDO selectivity, due to the occurrence of the glycerol dehydration–hydrogenation step (acrolein route), yielding 1-propanol and propane. Increasing Pt dispersion in the Pt/WO_x_/Al_2_O_3_ catalysts increased glycerol conversions; however, it contemporarily promoted the hydrogenolysis routes that lead to 1,2- and 1,3-PDO similarly. Instead, an augment of Pt metal content is favorable for hydrogenolysis route, thus significantly increasing the 1,3-propanediol production: probably a closer contact between Pt and WO_x_ promoted the hydrogenation of the intermediate secondary carbocation into 1,3-PDO.

**Figure 8:**
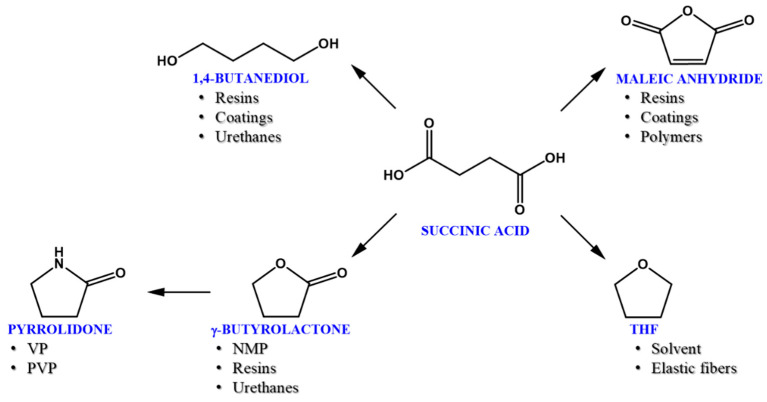
Possible routes by which renewable succinic acid can be further converted into value added products under heterogeneous catalysis.

Among the possible strategies for converting renewable feedstock into high added-value chemicals, catalytic methodologies offer several advantages, particularly if we consider the process optimization. Within this scenario, one of the most promising involves the catalytic conversion of various “platform” molecules (i.e., glucose, fructose, 5-hydroxymethylfurfural (HMF), levulinic acid, glycerol, 2,5-furandicarboxylic acid and 3-hydroxypropionic acid), frequently being obtained via either the fermentation or controlled depolymerization of cellulose. Some examples of such platform molecules include glucose, fructose, 5-hydroxymethylfurfural, 2,5-furandicarboxylic acid, 3-hydroxypropionic acid, levulinic acid and glycerol. Within this platform, succinic acid represents an interesting C4 substrate, due to the numerous derivative products that can, in principle, be obtained from it (Figure 8).

In the beginning, succinic acid was produced through the hydrogenation of butane-derived maleic anhydride; however, nowadays it is mostly being produced by several companies via the microbial fermentation of glucose on a kiloton scale. In a recent paper, optimal performance in terms of selectivity, activity and recyclability was observed using Pd/Al_2_O_3_ catalyst, able to promote the selective hydrogenative lactonization of succinic acid to γ-butyrolactone, having > 90% of selectivity and high levels of substrate conversion, under mild conditions [128]. Authors showed that the catalytic activity strongly correlates with the type of support and Pd particles size. An increased lactone selectivity was observed using Pd as active metal in comparison with other metals (e.g., Ru): this peculiar behavior was tentatively attributed, by the means of in situ diffuse reflectance infrared Fourier transform spectroscopy (DRIFTS), to the higher ability of Pd in the activation of carboxylic acid moiety of the substrate, thus facilitating the lactonization of the molecule.

An interesting analysis on Ag clusters supported on Al_2_O_3_ as effective heterogeneous catalysts for several green organic transformations, such as: (i) oxidant-free dehydrogenation of alcohols to carbonyl compounds, (ii) coupling of alcohols with amines to form amides and H_2_, (iii) N-alkylation of anilines with alcohols, (iv) C–C cross-coupling reaction of alcohols, (v) selective hydrogenation of nitroaromatics, and (vi) the direct synthesis of *N*-substituted anilines from nitroaromatics and alcohols, was published, with an accurate study on the effects of Ag particle size and acid-base character of support oxides on the reaction performances [129]. The relationship between structure and catalyst activity showed similar tendencies for all reactions: metallic silver clusters with a smaller size coupled with acid-base bifunctional nature of the support oxide are preferable in order to achieve the best reaction efficiencies, in the presence of suitable acidic or basic additives. Authors suggested that cooperation between acid base pair sites on the support surface and coordinately unsaturated Ag sites of Ag clusters, taking place at the metal–support interface, is a pivotal concept for designing Ag cluster catalysts for the above mentioned highly atom-efficient cited reactions.

Carbohydrates from renewable sources are considered a sustainable feedstock for green chemistry-based technologies as they represent about 75% of the renewable biomass produced every year. Thus, C_5_ and C_6_ sugars, of which glucose, fructose, and xylose are the principal ones, are readily available biomass primary compounds (Figure 6). In a recent paper, the direct dehydration of D-xylose to furfural was realized with the use of a biphasic liquid system containing toluene and water, working with a series of silica-based MCM-41-supported niobium-oxide catalysts obtained through an impregnation method [130]. Indeed, niobium containing catalysts was demonstrated to be active in reactions such as the dehydration of saccharides. The catalytic activity was attributed to the presence of silica-supported niobium species and increased along with niobium-oxide content. Furthermore, the furfural yield diminished for the highest niobium-oxide loading (up to 33 wt%), showing that the most active niobium-oxide species are the ones more dispersed on the silica support. A conversion of 74.5% and a furfural yield of 36.5% was reached after optimization of the experimental conditions, with the catalyst having a nominal 16 wt% of Nb_2_O_5_ (MCM-Nb_16_).

The heterogeneous catalysts were recyclable for a minimum of three reaction runs and they do not need an intermediate calcination step to remove carbon deposits for their reactivation. Interestingly, the addition of NaCl to the reaction system significantly increased the furfural yield, probably as a consequence of the promotion of 1,2-enediol formation from xylose acyclic form in the presence of chloride ions, and also for the increase in the partition coefficient, R (defined as the ratio between furfural concentrations in the organic over the one present in the aqueous phases) for the biphasic system toluene/water, after the addition of NaCl.

**Scheme 6:**
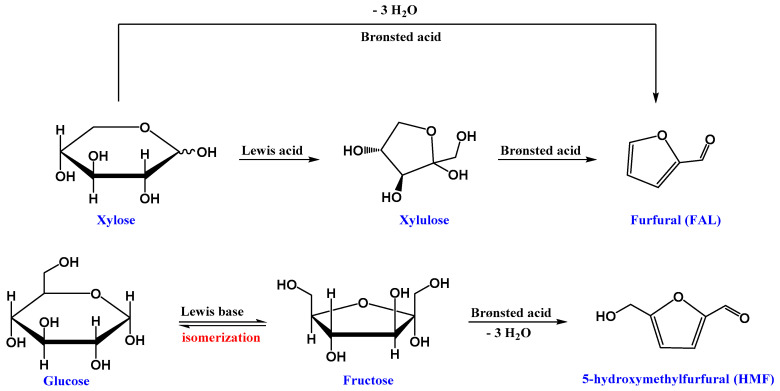
Schematic routes toward furfural (FAL) or 5-hydroxymethylfurfural (HMF) from different sugars.

In another study, the heterogeneous catalyst based on silica-supported phosphotungstic acid (PTA) was used for the preparation of HMF, starting from glucose (Scheme 6) in acetone/water (1:5, v/v) solvent, FAL being the main observed by-product which increased with the increasing of reaction time [131]. The silica support was previously derivatized with aminopropyltrimethoxysilane, in order to improve its adhesive efficiency, prior to the PTA loading. The HMF yield of 78.31% obtained with this PTA-based catalyst was fairly better than the one obtained with the previously reported heterogeneous catalysts, even in comparison with homogeneous FeCl_3_ and CrCl_3_ catalysts, in ionic liquids.

An interesting and versatile synthetic strategy was recently reported having in mind the preparation of multifunctional heterogeneous catalysts in which different active sites are separately activated by discrete functional groups present on the catalyst itself, to be used for sequential multistep biomass conversion. In this way, thermally robust sulfated zirconia (SZ) monolayers with tunable Lewis (from uncoordinated Zr^4+^ defective centers) and Brønsted (from coordinated sulfonate groups) acid sites were first grown onto nanostructured SBA-15 silica (named 2SZ@SBA-15), via sequential grafting and hydrolysis cycles using zirconium isopropoxide as precursor, followed by a sulfate-induced sintering procedure [132]. The tailoring of the right balance of Brønsted/Lewis acidity may provide the catalyst with either Lewis acid sites, necessary for glucose-to-fructose isomerization, or Brønsted acid sites, optimized for the subsequent fructose to HMF conversion. Then, the authors further used a grafting method to functionalize the 2SZ@SBA-15 hybrid with a base (–NH_2_), an acid (–SO_3_H) group, or both acid and base (–SO_3_H and –NH_2_) functional groups (namely, 2SZ@SBA-15-SO_3_H, 2SZ@SBA-15-NH_2_ and 2SZ@SBA-15-SO_3_H-NH_2_, respectively). The activities for all SBA-15 supported catalysts were assessed toward the multistep cellulose to HMF conversion, using an ionic liquids-based system, implying a series of cascade processes involving a decomposition step to glucose, an isomerization reaction to fructose and the fructose triple dehydration step with three molecules of water to HMF (Scheme 6). Having in mind the aim of unraveling the specific role of the catalysts in the sequential processes of cellulose to HMF conversion, the prepared materials with different Brønsted/Lewis acidity and basicity were employed in the reactions using fructose, glucose or cellulose as starting materials. The results of catalytic tests showed that Brønsted acid catalysis is the rate determining step for the oligomer-to-glucose depolymerization process, and that the presence of both Lewis acid and base catalysis plays a dominant role in promoting the isomerization of glucose-to-fructose. Noteworthily, under optimized conditions, a remarkable 54.0% of selectivity united to a HMF yield of 42.2% can be achieved from cellulose, with both acid-base functionalized catalysts such as 2SZ@SBA-15-SO_3_H-NH_2_. The catalytic system can be easily recovered and reused at least five times without a significant loss of activity, probably due to the retention of an ordered pore network with uniform and narrow mesopores in the support phase.

In an effort to find alternative strategies for biomass wastes valorization, recently, importance was given to the reuse of agricultural waste materials such as rice husk (RH), also with the aim of pollution reduction. RH is the outer part of rice produced during the rice refining process, containing a significant amount of amorphous SiO_2_. Silica extracted from RH showed good behavior as a catalyst support for the synthesis of fine chemicals [133]. Similarly, a series of highly mesoporous copper catalysts (5–20 wt%) supported on RH-derived SiO_2_ were prepared via the sol-gel technique at room temperature [134]. The activity of the copper-supported catalysts was tested in the liquid-phase oxidation of phenol using H_2_O_2_ as oxidant, obtaining catechol (CAT) and hydroquinone (HQ) as the only products. Excellent activity was reached in the case of 10 wt% copper catalyst and decreased with further increasing of copper loading, the latter being a robust and recyclable system which could be regenerated several times without significant loss in the catalytic activity.

#### 3.1.3. Ceria

With the advantages of reversible Ce^3+^/Ce^4+^ redox pairs, tailorable oxygen vacancies, and surface acid−base properties, ceria-based catalysts are actively investigated in the fields of catalytic organic synthesis [135]. This is the reason why, in recent years, several reviews on special organic reactions over CeO_2_-based catalysts were reported, such as transformations of nitriles and amides [136], CO_2_ conversion with alcohols or amines [137,138]; sustainable oxidation processes [139] and transformations of biomass, its derivatives, and downstream chemicals [140].

Indeed, reducible oxides, i.e., CeO_2_, are not only an excellent metal active phase supports but may also influence the intrinsic catalytic activity via metal–support interactions. In particular, cerium dioxide has attracted considerable attention, owing to its unique properties, such as high oxygen mobility and high oxygen storage capacity (OSC), enhanced thermal stability, and exceptional reducibility driven by the formation of either surface or structural oxygen vacancies, through the rapid transition between Ce^3+^ and Ce^4+^ [141,142]. In addition, apart from bare ceria’s exceptional properties, its combination with transition metals (as dopants or as supported active phases) improves catalytic performances of the overall catalysts, owing to the synergistic effect between the metal phase and the support, related to electronic, geometric and bifunctional interactions [41,143]. Considering that CeO_2_ is currently employed as efficient oxygen pump, quickly donating its lattice oxygen atoms for oxidation reactions, then the reduced cerium oxide may be subsequently re-oxidized by O_2_; this facile O_2_ transport could bring significant improvements in catalytic systems. A previous work established that CeO_2_ nanoparticles are one of the best support materials for Au-catalyzed alcohol oxidation for the above mentioned reasons [144,145].

**Scheme 7:**
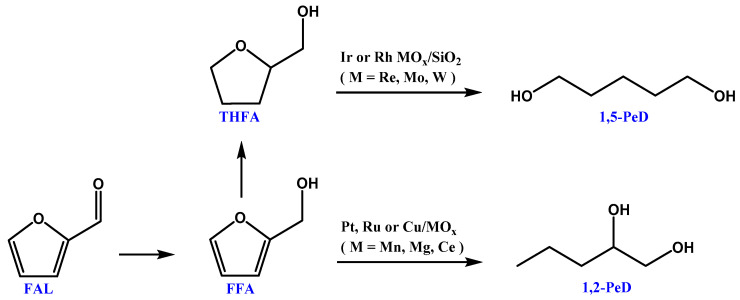
Reaction path of the hydrogenation and hydrogenolysis of furfural (FAL).

In a recent paper, it was reported that by supporting Ir on CeO_2_ as a highly active and selective heterogeneous catalyst for numerous transfer hydrogenation reactions (aerobic benzyl alcohol oxidation and cyclohexanone MPV hydrogenation) [146]. The activity of this catalytic system was related either to the properties of the support, namely the transport oxygen ability CeO_2_, or to the nanoparticulate Ir_2_O_3_ centers, that is the major active species, preferentially formed through the high-temperature (400 °C) reduction of the as-prepared catalyst. Careful mechanistic investigations allowed authors to hypothesize that the activity of the catalyst is due to its ability of forming metal hydroxide species that are afterwards transferred either to a molecular oxygen or to a ketone, respectively. In particular, the catalyst was able to perform such reactions at temperatures lower than 100 °C, without stoichiometric quantities of a base (i.e., base-free) and with O_2_ or air at ambient pressure. Nevertheless, the main drawback for the catalyst deactivation, was attributed to the radical-based formation of benzoic acid, as by-product. Indeed, the coordination of the carboxylate to the metal oxide center significantly decreased the catalyst activity. Furthermore, the direct hydrogenolysis of biomass-derived furans to high value-added alcohols was successfully conducted over a highly active and stable Pt/CeO_2_ heterogeneous catalysts [147]. In this case, CeO_2_ as support showed superior catalytic performances compared to other supports such as silica, alumina or MgO, in terms of selectivity toward 1,2-pentanediol (1,2-PeD, Scheme 7), thus confirming that supports play a fundamental role for the ring-opening of furfural: authors suggested that the basic sites of appropriate strength, in CeO_2_ could play a beneficial role on the selective ring-opening of furfural alcohol (FFA) toward the 1,2-PeD formation, in comparison to 1,5-PeD (Scheme 7).

**Figure 9:**
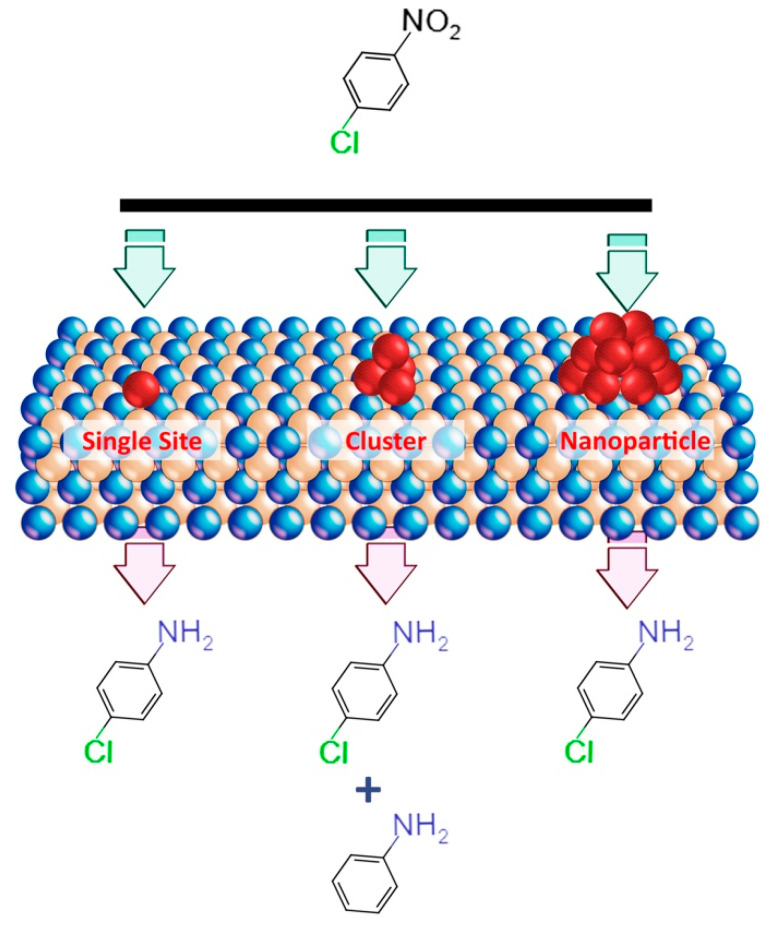
Selectivity change in para-chloronitrobenzene (p-CNB) hydrogenation to para-chloroaniline (p-CAN) and aniline, on selected Pt/CeO_2_ catalysts with different Pt loadings.

Many studies have demonstrated that isolated atoms of platinum [148,149,150], palladium [151,152], or ruthenium [153,154], supported on oxides or carbons, are able to efficiently catalyze a number of important reactions with high selectivity. Nevertheless, the deep understanding for their extraordinary performances is not, so far, well documented. Recently, by means of a series of experiments coupled with density functional theory (DFT) studies, the unusual size dependence with respect to selectivity in para-chloronitrobenzene (p-CNB) hydrogenation reaction performed by the means of CeO_2_-supported Pt catalysts was investigated [155]. Despite the investigated substrate not being directly derived from biomass sources, it can serve as a model that can be directly translated towards biomass-derived molecules. It was found that the selectivity toward para-chloroaniline (p-CAN) vs. aniline formation, changed drastically with Pt loading (Figure 9).

DFT data evidenced that the differences on the main orbitals among Pt sites of diversely coordinated environments had a huge influence on the interaction modes with the C–Cl bond and was a pivotal factor to determine p-CAN selectivity in p-CNB hydrogenation. High-coordinated platinum sites with a large steric hindrance were quite inactive towards dehalogenation, although the low-coordinated ones having less steric hindrance displayed noticeable activity towards the C–Cl bond cleavage. Platinum single-site, Pt_1_/CeO_2_, formed through occupation of a vacancy on ceria surface by a single Pt atom, could be seen as an alternative to high-steric-hindrance environment sites, necessary to achieve either high selectivity or atomic economy in the hydrogenation of p-CNB.

#### 3.1.4. Layered Double Hydroxides (LDH)

**Figure 10:**
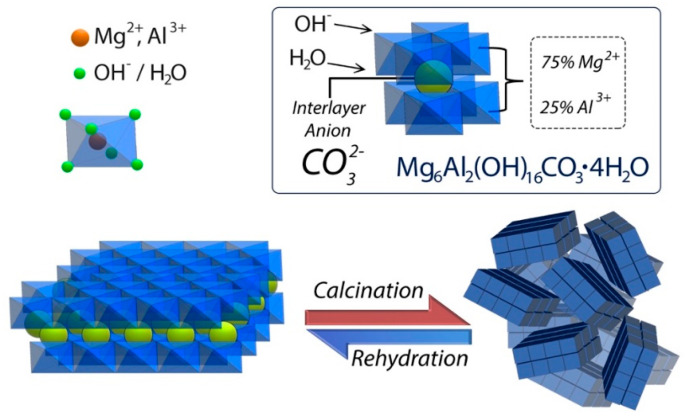
Illustration of the crystal structure, calcination of Mg–Al layered double hydroxide (LDH) and rehydration of the calcined LDH.

LDH, also known as hydrotalcite-like compounds or anionic clays, are basic, homogeneous, mixed metal hydroxides possessing a lamellar structure, formed by positively charged mixed metal hydroxide layers and negatively charged anions, with water molecules in the interstitial layers (Figure 10). The general formula is often depicted as [M^2+^_(1−*x*)_ M^3+^_(*x*)_ (OH)_2_]*^x^*^+^[A*_x/n_*]*^n^*^−^·*m*H_2_O, where [M^2+^_(1−*x*)_ M^3+^_(*x*)_ (OH)_2_]*^x^*^+^ represents the Brucite-like layer, and [A*_x/n_*]*^n^*^−^·*m*H_2_O represents the interlayer composition, with the divalent cation that can be Mg^2+^, Ni^2+^, Mn^2+^, Co^2+^, Fe^2+^, Zn^2+^, Cu^2+^, or Ca^2+^, while the trivalent cation can be Cr^3+^, Co^3+^, Mn^3+^, Fe^3+^, Al^3+^ or La^3+^, and A^n−^ representing the anion [156]. Such materials can be prepared through precipitation from soluble salt precursors or by ion exchange. LDH thermal decomposition brings to the formation of mixed oxides, exhibiting an interesting property defined as “memory effect”, by which original layered structure can be restored whenever the mixed oxides come in to contact with aqueous solutions containing different anions or even with vaporized water (Figure 10).

**Scheme 8:**
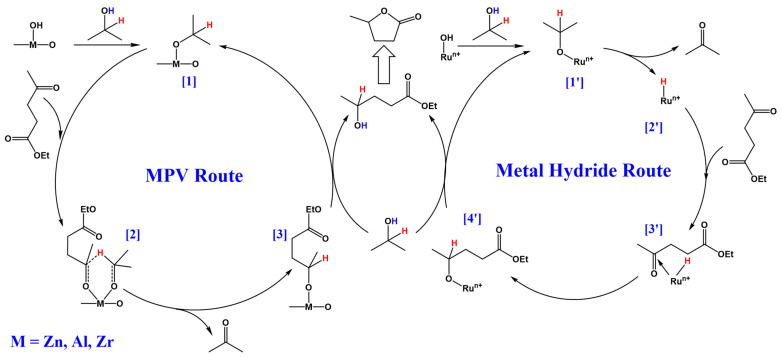
Proposed possible mechanisms for the transfer hydrogenation of ethyl levulinate (EL) to γ-valerolactone (GVL) over the Ru/ZnAlZr-LDH (Meerwein-Ponndorf-Verley (MPV)).

Due to the unique properties of layered double hydroxides, including (i) tunable basicity and acidity, (ii) thermal stability, (iii) anion-exchange ability of the interlayer space, (iv) cation-exchange ability of the Brucite-like layer and (v) large adsorption capacity, they could potentially offer significant aid for the conversion of biomasses (Figure 2), either directly as catalysts or as active supports for multifunctional catalysts. For the above-mentioned reasons, LDH derived materials were investigated as catalytic promoters in several well-studied routes (e.g., isomerization, oxidation, dehydration, hydrogenation, steam reforming, aldol condensation and depolymerization), representing the key steps in the catalytic conversion of biomass. To this end, some excellent general reviews describing their applications in the field of catalysis were recently published [157,158,159,160,161,162,163]. As a specific example of biomass valorization, the use of a ruthenium-based catalyst, namely Ru/Zn-Al-Zr layered double hydroxide (Ru/ZnAlZr-LDH), in the synthesis of γ-valerolactone (GVL) via transfer hydrogenation of biomass-derived ethyl levulinate (EL), employing 2-propanol as hydrogen donor, was recently published (Scheme 8) [164].

The combination of Ru species and ZnAlZr-LDH matrix, modified the electronic properties of Ru^3+^ species, whose electron-rich nature as Ru(OH)_3_ was confirmed by XPS analysis. This heterogeneous catalyst showed very high catalytic activity, in comparison to other Zr- or Ru-based catalysts, affording high GVL yield of 98%, only within 10 min of the reaction. The presence of a large amount of surface -OH groups together with highly dispersive electron-rich Ru species is beneficial for the activation of hydroxyl groups in 2-propanol and carbonyl species in EL substrate, thus greatly facilitating the formation of activated six-membered ring transition state (Scheme 8—Structure 2) and active ruthenium-hydride species during transfer hydrogenation processes: this may be on the basis of the high activity observed, as described by the authors.

#### 3.1.5. Zirconium-Based Oxides and Other Types of Oxide Supports

Most of the biomass conversion processes are often governed by carbocation chemistry, requiring acid catalytically active sites. Solid acid catalysts are about the most popular heterogeneous catalysts and are largely employed in biomass conversion. Among several other types, ZrO_2_-based catalysts have received large attentions both as a catalyst itself and as support, due to their desirable features and catalytic properties [165,166,167]. Due to their strong acidity, high catalytic performances, easy tuning, high hydrothermal stability, easy recoverability and reusability, and environmental friendliness, it is believed that zirconia and zirconia-based catalysts (such as, for example, zirconium containing perovskites [168]) represent one of the best multifunctional solid acid catalysts for many acid-catalyzed biomass conversions [169]. A recent paper reported a Zr-MCM-41 and amberlyst-15 mixed catalyst, with a highly effective catalytic hydrogenation rate, was employed for the acid-catalyzed conversion of furfural to biofuel alkyl levulinate (replacing external H_2_ by means of isopropanol as the hydrogen donor) [170].

**Scheme 9:**
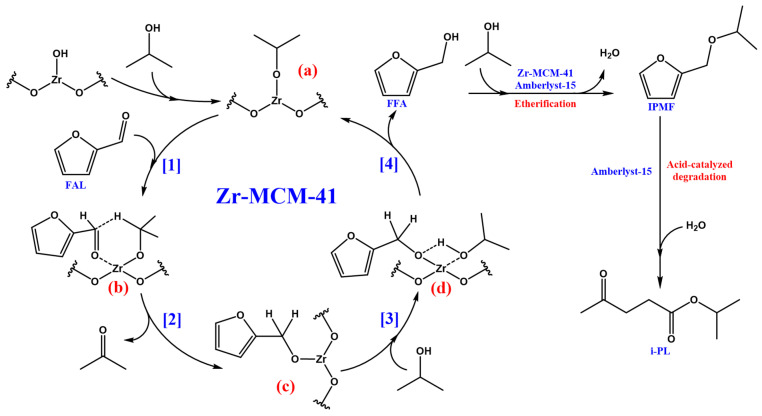
Proposed mechanism for isopropyl levulinate (i-PL) synthesis from furfural using isopropanol as the hydrogen donor by combing Zr-MCM-41and Amberlyst-15.

Authors proposed a tentative reaction mechanism illustrated in Scheme 9, where the unsaturated Zr^4+^ of Zr-MCM-41 binds to isopropanol, forming Zr-bound isopropoxide (species a) that then coordinates with the carbonyl oxygen of furfural, newly yielding to a six-membered ring transition state (Scheme 9—Structure b). The hydride in species b subsequently transfers from alkoxide to the carbonyl group, forming the intermediate species c. Afterwards, a new isopropanol molecule will coordinate with species c, forming species d, subsequently dissociating to release FFA. It will then undergo etherification with isopropanol, forming the main intermediate 2-isopropoxymethylfuran (IPMF). The latter, after being subjected, respectively, through the processes of protonation, elimination, and isomerization catalyzed by amberlyst-15, is then converted to isopropyl levulinate (i-PL). By the means of this synthetic route, an optimized alkyl levulinate final yield of 85.3% was declared, in a one pot reactor at 130 °C for 24 h. When zirconia is present as dopant in MCM-41, the solids display all the common features of undoped MCM-41, but also introduces Lewis and Bronsted acid sites, thus largely widening its applications field [171]. 

Concerning zirconium mixed oxides, there are a couple of noteworthy pieces of work related to Zr-based perovskites employed as supports in biomass conversion reactions. Girard and co-workers prepared a series of zirconium-based catalysts impregnated with Pt, in the 0.5–0.7 wt% range, that were successfully tested in the syntheses of short-chain aliphatic glycols (C_2_-C_6_) derived by cellulose depolymerization. In particular, CaZrO_3_, SrZrO_3_ and BaZrO_3_ solids having a perovskite structure presented a similar basic character with respect to zirconia. Pt nanoparticles deposited over perovskites were shown to be able to obtain a much higher cellulose conversion in comparison to the case of Pt/ZrO_2_ based systems (97% for BaZrO_3_ against 56% for ZrO_2_). Selectivity was further improved by employing CeCl_3_ as homogeneous co-catalysts reaching, respectively, different yields in terms of ethylene glycol (21.7%), propylene glycol (19.2%) and 1,2-hexanediol (2.5%) [172]. Keller and collaborators instead used ZrO_2_ as support for either La, Sr or Fe metal species and employed the synthesized materials as catalysts for the reforming reaction of biomass tar model molecules, such as benzene and ethylene. They found that the reaction environment induces the formation of different La_2_Zr_2_O_7_, SrZrO_3_ or LaFeO_3_ phases (depending on the metal phase deposited on zirconia) with perovskite structure, on the surface of the support, which are the active ones involved in the benzene and ethylene conversion [173].

As a matter of fact, research results indicate that the surface properties and structure of the supports act remarkably in enhancing reaction selectivity, while strong metal–support interaction and the metal nanoparticles high dispersion are responsible for the high catalytic performance and selectivity. Additionally, the loading of noble metals as dopant on oxide/hydroxide supports has emerged as an efficient strategy to reduce the production cost of catalysts; nevertheless, it must be underlined that the support may act either as poisoner or as promoter. Thus, the choice of the most suitable catalytic support is an important point in order to obtain affordable, stable and efficient catalysts.

An interesting report based on the comparison of the catalytic activity of vanadium oxide (VO_x_) supported on different metal oxides such as TiO_2_, Al_2_O_3_, Nb_2_O_5_, ZrO_2_, and MgO, having a wide range of acid–base and redox properties, related to the HMF oxidation toward 2,5-diformylfuran (DFF), was published [174]. The latter compound is known as a versatile reaction intermediate for the synthesis of pharmaceuticals, Schiff bases, and antifungal agents, as well as of polymers for various applications [175,176]. Authors examined the effects of the VO_x_ surface densities and support features on the reducibility, activity, and selectivity of the active VO_x_ domains. Basically, these structures evolved from monovanadate to 2D polyvanadate structures, thus raising VO_x_ sites surface density, finally bringing to a crystalline V_2_O_5_ clusters, when the surface density of such species grows above one-monolayer capacity. Below that level, good VO_x_ surface densities couple with easily reducible supports (such as TiO_2_ and ZrO_2_) brought to higher reactivity and reducibility of VO_x_ domains. Moreover, polyvanadates and V_2_O_5_ clusters covering the surface of supports with higher acidity favored the formation of DFF. Finally, the correlation between the reducibility and reactivity, coupled with kinetic studies, suggested that the 5-hydroxymethylfurfural oxidation to 2,5-diformylfuran occurs via redox mechanism, involving the V^5+^/V^4+^ redox cycles, followed by the reoxidation of V^4+^ to V^5+^ by means of molecular oxygen as the rate-determining step.

For other kinds of reactions, the need of a more basic support can be required in order to improve the catalytic activity, such as the oxidation of chitin monomeric units in basic solvents performed with gold nanoparticles. As mentioned in Section 1.2., chitin is composed by glucosamine, galactosamine, mannosamine and their derivatives, acting as a precious source of aminoacids that are employed in the medical field and in the food transformation industry. Ohmi and collaborators have demonstrated that 3 nm textured gold nanoparticles deposited onto basic supports, such as hydrotalcite (HT: Mg_6_Al_2_(OH)_16_CO_3_∙*n*H_2_O), MgO or CaO, are able to catalyze aqueous oxidation of chitin constituents, affording up to a 93% amino acids yield, as in the case of the Au/MgO system [177].

### 3.2. Metal Organic Frameworks

**Figure 11:**
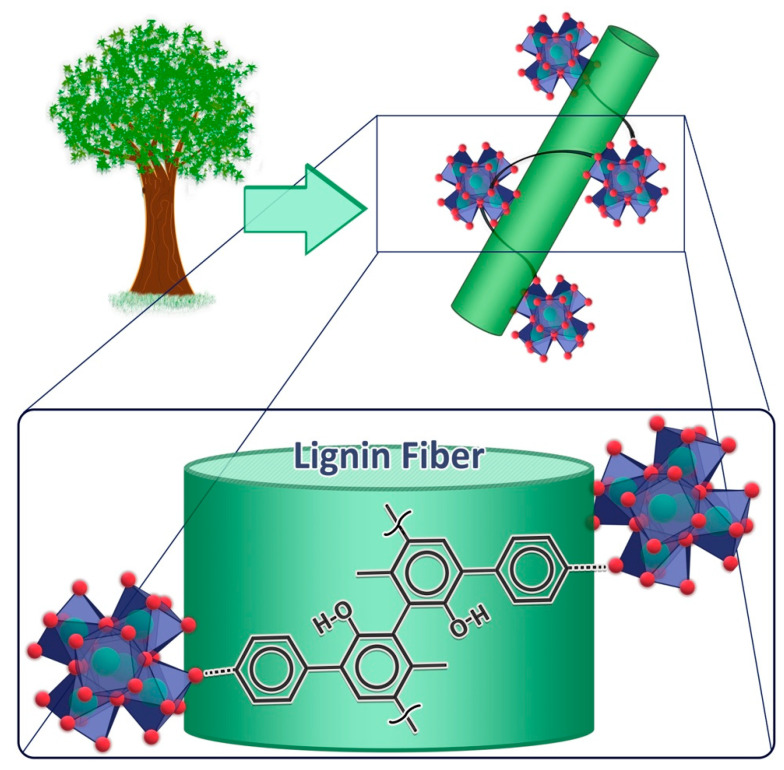
Metal organic framework (MOF)-lignin hybrid material inspired by linear coordinated MOFs synthesized by Jiang and collaborators [189].

Single-site heterogeneous catalysts have aroused great interest in several catalytic reactions due to their uniform and distinct geometric and electronic structure. Among the various porous supports and metal-organic frameworks (MOFs), through molecular-level structure control and easy modularity in common 3D or less common 2D and 1D arrangements (Figure 11) are offered versatile opportunities for supporting isolated single site catalysts. The MOFs used either as support for single-site catalysts [178] or for in situ grown nanoparticles [179] were successfully constructed using different strategies and exhibit excellent catalytic performances. The highly ordered arrangements of organic linkers and metal nodes as well as the well-defined nanopore structures of the MOFs make them ideal substrates for supporting atomically dispersed metal sites without the need for bulky, elaborately designed linkers. Moreover, naturally present functional groups of the linkers may act as further functionalization points for more specialized anchoring groups for single site catalysts [180]. The high surface area generates a very high density of catalytic sites, allowing greater possibilities of contact with substrates and producing catalysts with high activity and excellent steric selectivity, tunable by changing linkers shape and length in the synthesis steps [178]. The range of reactions that can be performed with MOF-based catalysts is almost as wide as their synthetic tunability: industrially relevant reactions involving commodities production (butenes syntheses for synthetic rubber production) [181]; environmental remediation reactions with greenhouse gases abatement (CO_2_ reduction through thermal- [182,183] or photo- catalytic processes [184]); photocatalytic [184] and electrocatalytic [185] hemi-reactions for fuel cells processes; and reactions for fine chemicals production [186]. Apart from these cases, there are only a few (yet remarkable) examples of MOFs employed as supports for catalysts employed as biomass valorization processer finalized to the production of higher added-value molecules.

Xinle Li et al. developed Pd nanoclusters (NCs) encapsulated in differently supported UiO-66 MOFs, with differently functionalized linkers (phenyl, amino-phenyl or metoxy-phenyl), generally employed to catalyze the aerobic reaction between benzaldehyde and ethylene glycol. In particular, the direct functionalization of the linkers can finely tune the product distribution (cyclic or linear) of this reaction. They observed that amino-functionalized Pd@UiO-66 favors the formation of cyclic acetal, while Pd@UiO-66 and metoxy-functionalized Pd@UiO-66 have a greater selectivity for the ester product. DRIFTS studies confirmed that −NH_2_ groups coordinate the Pd surface and work as electron-donors to the Pd NCs. The NH_2_-Pd interactions are able to lower the oxidation capacity of encapsulated Pd nanoclusters, giving a high selectivity towards acetal for the reaction performed with Pd@UiO-66-NH_2_. This strategy can be extended to the hydrogenation of furfural in the gas phase, and the modification of the linker in the MOFs is also decisive for the distribution of the product [187].

Yasutaka Kuwahara et al. studied the γ-valerolactone formation reaction starting from biomass-derived levulinic acid and its esters via a hydrogenation transfer catalyst onto sulphonic acid functionalized Zr-based UiO-66 MOF. Comparative experiments, combined with the characterization techniques, demonstrated that the catalytic activity is given by the cooperative effect between the Zr_6_O_4_(OH)_4_ clusters (acting as Lewis basic sites) and −SO_3_H species acting as Brönsted acid sites. These moieties, positioned in a narrow space and adjacent to each other, catalyze the hydrogen transfer reaction of levulinic acid and its esters, favoring the subsequent intramolecular dealcoholization to obtain γ-valerolactone. The catalyst is also reusable, without any appreciable loss of activity and selectivity [188].

Hong Jiang et al. report the synthesis of highly stable chiral MOFs based on Zr (IV) with different topologies as supports for iridium complexes. Five chiral Zr-MOFs with different topologies were synthesized using enantiopure 1,1′-biphenol-derived tetracarboxylate linkers and progressively larger Zr6, Zr9 or Zr12 clusters. All the obtrined MOFs display a high chemical stability in water, in strong acidic and weak basic aqueous solutions. The MOF, which underwent post-synthetic modifications with P(NMe_2_)_3_ and [Ir(COD)Cl]_2_, was highly efficient for the hydrogenation of α-dehydroamino acid esters with an enantiomeric excess up to 98%. The incorporation of Ir-phosphorus catalysts into the Zr-MOF structure leads to excellent results by improving their chemical stability and stereoselectivity [189].

Ning and collaborators synthesized a series of UiO-66 MOFS, by changing the nature of the linkers, moving from phenyl to −NH_2_, −NO_2_, −COOH and −NH_3_Cl phenyl-functionalized linkers. Gold nanoparticles were grown by the impregnation of MOF support with HAuCl_4_, followed by in situ reduction processes with different reducing agents. The produced catalysts were tested for oxidative valorization of furfural with methanol and ethanol. While in the first case, the traditional UiO-66 MOF performs perfectly in the production of methyl-2-furoate, with 100% conversion and selectivity. In the second case, the functionalization is necessary to pass from 44.1% conversion with 40.2% selectivity towards furan-2-acrolein for classical UiO-66 MOF, to 77.2% conversion and 95.3% selectivity towards furan-2-acrolein for the −NH_3_Cl functionalized UiO-66 [190].

Lignin-derived aryl ethers selective hydrogenolysis reaction under sustainable temperature and pressure conditions, is of pivotal importance in order to cover the increasing global demand of fuels derived from largely available biomass sources. Moreover, the selective hydrogenolysis reaction demands quite fine modulation of active sites at the surface of the catalyst, necessary to attain specific activity and selectivity. In this context, the latter type of reaction applied for benzyl phenyl ether conversion to phenol and toluene, performed in MeOH and H_2_O using very low temperature and hydrogen pressure using a Pd nanoparticles-decorated Ce-BTC (benzene-1,3,5-tricarboxylate) metal organic framework (MOF), has been reported [191]. In the literature, it is possible to find that supported small-sized Pd nanoparticle catalysts are pretty efficient in aryl ethers selective hydrogenolysis. Nevertheless, the high surface energy possessed by small-sized metal nanoparticles promotes metal aggregation under reaction conditions. Frequently, the use of a sacrificial support may slow down the coalescence process, as demonstrated in this case by means of Pd NPS loading onto Ce-BTC MOF. The latter showed two-fold higher catalytic activity in comparison to the system formed by Pd nanoparticles-decorated ceria, under identical reaction conditions. A rational was established by authors through XPS and DFT studies, by which it was possible noticing that the concentration of Pd^0^ species (it is well established the ability of Pd^0^ to dissociatively adsorb molecular hydrogen, forming Pd–H active species, exhibiting good activity towards C–O hydrogenolysis) is higher in Pd/Ce-BTC rather than in Pd/ceria. Additionally, the adsorption energy of benzyl phenyl ether onto Ce-BTC is higher than the one for Pd/ceria; conversely, the desorption energy of phenol over Ce-BTC is lower with respect to the one over CeO_2_: all these factors could be responsible for the increased activity showed by the Pd/Ce-BTC system.

### 3.3. Nanoparticles

**Figure 12:**
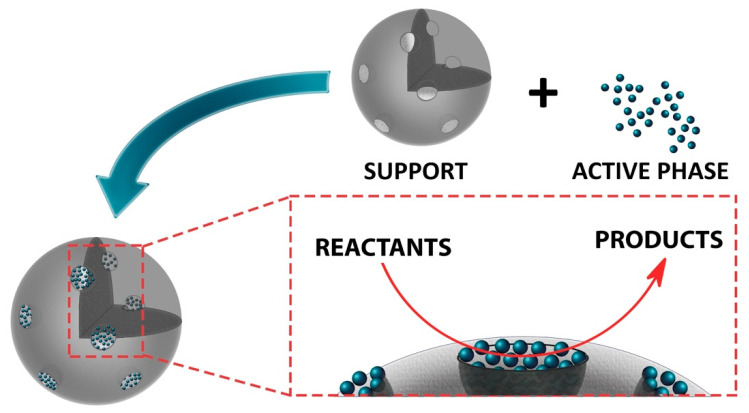
Example for hybrid nanoparticles in reaction environment, in which the particle itself acts as support for smaller clusters on the surface which are active in the catalytic processes.

The heterogeneous catalytic systems most commonly employed for advanced chemical reactions are developed in three dimensions and have different components [192], including active metal nanoparticles and metal oxide supports [193]. However, the tendency nowadays is more and more to consider nanoparticles as a support platform for heterogeneous catalysis, being considered the bridging point between homogeneous and heterogeneous catalysis [194]. The first type of nanoparticles might be considered as traditional supported catalysts are hybrid metal nanoparticles, which are conceptually very close to real catalysts, considering the main metal as the bulk support and the doping one as the active phase (Figure 12). Their controllable morphology makes them suitable for investigating in detail the relationship between structure and catalytic properties [195]. There are several typical structures of hybrid metallic nanoparticles that can be used to conduct organic catalysis and photochemical reactions, and the most common are core-shell systems [196]. Core-porous shell metal-silica (or other metal oxides) nanoparticles, show extremely high thermal and chemical stability, coupled with remarkable resistance to sintering. The morphology of the metal cores and the density of the pores of the cavity of the shells can be suitably modulated to optimize the catalytic activity and the diffusion rates of the reactants, with high activity and no barrier to diffusion of reactants and products [197,198]. These nanostructures are used as effective catalysts for various organic and gas phase reactions, including hydrogen transfer [199], Suzuki coupling [200,201,202], and methane reforming [203]. Differently from the core-porous shell structures, other properties given to these hybrid systems might be magnetism (given by the core) and light absorption (provided by the shell layer). In the latter case, there are examples reporting metal core-semiconductor shell nanostructures that could behave as effective photocatalysts for CO-oxidation reactions [204,205]. The diffusion limit of the reagents can be exceeded by an inverted core-shell structure. Although these hybrid metal nanoparticles now resemble real heterogeneous catalytic systems, numerous studies remain to be performed. The most significant argument in the field of catalysis is the achievement of regio- and stereo-selectivity [206,207]. Another limitation of current research is the generation of a charged surface in a heterogeneous system. All metals used so far for nanoparticles in organic reactions have neutral surfaces, although most organometallic species exhibit formal charges, which might have serious concerns about the contribution of such model catalysts to industrial reality. Even if these catalysts cannot be directly applied to industry, related research must at least provide a better understanding of the behavior of the catalysts themselves and develop systems with which to enhance the catalyst in such a way as to mimic real systems [195].

In those cases in which shell modification is not enough to confer the desired properties to the nanoparticle-supported catalyst, the use of surface organo-metallic chemistry (SOMC) for the introduction of active metal sites can be of great importance [208,209], that combined with thermolytic molecular precursors (TMPs) that lose their organic fractions fairly easily after heat treatment provides access to isolated supported metal sites with the same oxidation state as the precursor. The resulting surface species show a strong resemblance to the active sites of the metals supported onto oxides and also contain a higher value in proportion of active sites which makes it possible to study their structure–activity relationship [210]. They are thus ideal models for the class of industrial catalysts used in applications such as the epoxidation of olefins [211,212], metathesis of olefins [213,214], polymerization of ethylene [215,216,217] or dehydrogenation of light hydrocarbons [218,219,220]. Despite SOMC is an excellent tool to graft metal sites directly onto the surface of supports, this approach is mostly used to functionalize macro/microscopic crystals and not nanoparticle supports. The most common method employed for nanoparticles functionalization is the one developed for organometallic complexes anchoring onto Super Paramagnetic Iron Oxide Nanoparticles (SPIONs) surface: the desired complex is modified via reaction with a proper organosilane; afterwards, the Si-containing species is decomposed creating an oxygen bridge between the surface of the bare/silica-coated SPIONs and the metal-bearing molecule. The choice of such strategy is suggested by the ease of recovery of the catalyst and of the process for magnetic and silica-coated magnetic nanoparticles functionalization [221,222]. This peculiar strategy is used to deposit Schiff-bases and other chelating agents dedicated either to olefins epoxidation [223,224,225,226,227,228,229], sulfide oxidation [228,230,231], alcoholysis of epoxides [232], or homocoupling of terminal alkynes [233]. Although the large number of papers available, organometallic-functionalized magnetic nanoparticles are rarely employed for fine chemicals production through biomass valorization. However, there are some examples in which diversely functionalized magnetic nanoparticles act as supports for biomass conversion into fine chemicals.

Costa et al. deposited a ruthenium hydroxide layer on top of amino-derivatized iron -based silica coated magnetic nanoparticles (SCMNPs). Thanks to the amino groups of the support, they obtained a homogeneously dispersed amorphous layer of Ru(OH)_x_ on the surface of the catalysts, able to obtain carbonylic monoterpenoids (which are important molecules for cosmetics and pharmaceutical industries) in good-to-excellent yields starting from biomass-based monoterpenic alcohols, such as isoborneol, perillyl alcohol, carveol, and citronellol [234]. The work from Wang and collaborators used iron oxide SCMNPs as support for the anchoring of 12-tungstophosphoric acid (HPW) acting as acid catalysts, due to its Keggin-type structure [235]. They successfully used the obtained catalyst to produce 5-ethoxymethylfurfural from 5-hydroxymethylfurfural etherification reaction (with 83.6% yield) or directly from fructose (with 54.8% yield) [236]. Farzaneh and Rashtizadeh employed SCMNPs with iron oxide core properly functionalized with 3-aminopropyltrimethoxysilane (APTMS) to anchor a Schiff-base synthesized starting from histidine and glutaraldehyde. The formed support was used to load oxovanadium species starting from vanadyl sulfate, and the catalyst was tested in geraniol epoxidation with excellent performances [237]. The team of Yang and co-workers covered magnetite nanoparticles with a mercaptopropyl-containing periodic mesoporous organosilica (PMO) shell. The support was impregnated with Ru precursor, ending up in ruthenium nanoparticles stabilized by the presence of mercapto groups. The obtained catalyst showed good activity in direct conversion of cellulose to isosorbide [238]. Halilu and collaborators prepared a catalyst based on a magnetic Ni^2+^-substituted magnetite core, covered by a silica shell. Nickel substitution inside the magnetic inner part of the support happens during the Ni nanoparticles deposition process. The material was tested in the hydrogenation reaction of furfural to furfuryl alcohol [239]. The last case of a magnetic nanoparticles-based catalyst support that we report is from the work of Yuan and co-workers. They used chitosan-coated MNPs, exploiting the amino-functionalization to graft acrylate-derivatized metalloporphyrins on the surface of the support. The produced catalysts were tested for hydrolysis of corncob cellulose into 5-hydroxymethylfurfural (5-HMF), performing the reaction in various ionic liquids as solvents. The combination between 1-(8-mercaptooctyl)-3-methylimidazolium hexafluorophosphate ((moMIM)(PF_6_)) as solvent and porphyrin bearing a combination of Al^3+^, Cr^2+^ and Mg^2+^ ions as catalyst, provides 5-HMF yield of 66.6% [240].

Up to this point, we dealt with cases in which the supports designed to actively stabilize/coordinate the catalytically active species. Although these cases are quite numerous, they are not the only ones; indeed, also non-magnetic nanoparticles serve as catalytic supports in fine chemicals production from biomass wastes, even if with a lower number of cases. Yang et al. developed a nanospheric mesoporous silica (NS-MS) decorated with bimetallic oxides (Co–Mn), having a synergistic catalytic effect of Co–Mn bimetals in the base-free for the selective oxidation of 5-hydroxymethylfurfural (5-HMF) derived from biomass. In particular, by means of a modified micellar modeling, controllable quantities of Co–Mn bimetals were chemically inserted on the surface of the mold micelle and then in situ mesopores were generated by the thermal degradation of the micelle. The strong metal–support interaction established by the average effect of the counterions induced a high dispersion of the metal species. The relationship between catalytic activity and surface=active species was related to the selective oxidation process of 5-HMF [241]. Nano-silica is also used as support for anchoring ionic liquids on its surface to perform the dehydration reaction of fructose to obtain 5-HMF. In particular, the research group of Coutinho supported 1-(tri-ethoxy silyl-propyl)-3-methyl-imidazolium hydrogen sulphate (IL-HSO_4_) on the surface of silica nanoparticles. At optimized reaction conditions (fructose = 50.0 mg, catalyst = 40.0 mg, *T* = 130.0 °C, DMSO solvent = 0.5 mL), 99.9% fructose conversion and 63.0% 5-HMF yield were achieved when using the silica nanoparticles supports with the largest dimensions (~500 nm) in 30 min reaction [242]. Alternatively, Lee and Wu used mesoporous silica nanoparticles hybrid-functionalized with propyl-sulphonic groups and 1-ethyl-3-propylimidazolium chloride groups bearing Cr^2+^ ions. Such catalyst tested for fructose dehydration into 5-HMF, displayed conversion >99% with 72.5% furfural yield after 3 h reaction (in DMSO solvent at 90 °C) [243].

### 3.4. Two-Dimension and Carbonaceous Materials

**Figure 13:**
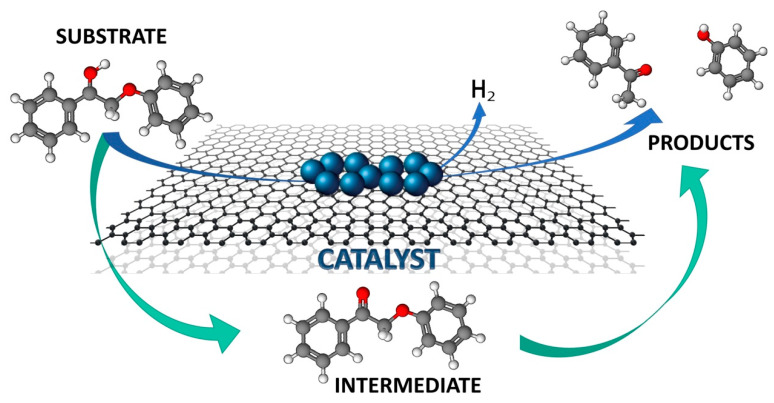
Schematization of a general reaction taking place on the surface of a generic 2D material.

Carbonaceous materials, such as activated carbons discussed in Section 2.1, possess an enormous surface area and exceptional chemical inertia. However, being most of the times natural in origin, their chemical composition is hardly controllable. Synthetic carbonaceous materials (such as graphenes) can be exfoliated from stacked graphite to obtain pure sp^2^ character, or they can be synthesized by pyrolysis of organic molecules onto flat surfaces (Figure 13). With the latter method it is possible to control the insertion of heteroatoms (the most common are B, N, O and S) to confer peculiar properties to the materials. Such properties can be exploited to better stabilize certain active phases or acting as directing agents for chemical reactions. It is shown that nitrogen-doped 2D carbon decorated with functional particles, such as hybrid metals NPs, are used for formic acid dehydrogenation, unsaturated bond hydrogenation and the production of high-value chemicals. The support based on carbon/nitrogen materials causes an adjustment of the electronic density of the metal center which directly causes an enhancement of the catalytic activity. The introduction into these supports and the modification of different metal particles, which leads to a rich biomass conversion chemistry, are thus being studied. Furthermore, carbon/nitrogen materials are easily applicable to biomass production for a combination of an efficient Mott-Schotty effect and diversity of metal sites [244]. Not only carbonaceous materials are interesting for such applications, also dichalcogenides are already exploited (especially MoS_2_) for example in the desulphurization reaction; however, the cases in which these materials are employed for biomass conversion are much fewer.

Shubo Tian et al. used two samples of single atomic site Ir supported on a defective metastable phase of titanium dioxide or on mesoporous graphitic carbon nitride, they demonstrated the effects of metal–support interactions on the regulation of geometric and electronic structures of the catalytically active central Ir species. Experimental results show that single atomic site Ir catalyst supported by the defective metastable phase of titanium dioxide exhibits high catalytic activity for hydrogenation of furfural to furfuryl alcohol, with 99% conversion, high selectivity and good stability. These results are better than those obtained with the Ir sample supported on carbon nitride and the Ir nanoparticles as such. The excellent catalytic performance of Ir supported on titanium dioxide can be attributed to the strength of the interactions between the active metal sites and the reactant molecules, with a synergistic action of the support. This work demonstrates that the properties of single atomic site catalysts can be modulated by metal-support interactions, and such a strategy could be used as an approach for designing new, more catalytically efficient catalysts [109].

Xin Jin et al. focused on a bimetallic nanocatalyst for the conversion of biomass-derived polyols into chemicals with wider applications for various reactions of industrial relevance. In particular, a new strategy is described to synthesize copper-based nanocatalysts on reduced graphene oxide supports. This method produces highly active Cu–graphene catalysts for converting biopolyols (glycerol, xylitol and sorbitol) to value-added chemicals, such as lactic acid and other useful co-products consisting of diols and linear alcohols. The addition of palladium in the traces of the Cu–graphene system leads to a tandem synergistic effect in which the hydrogen generated in situ from the polyols is used for the sequential hydrogenolysis of the starting material itself. Furthermore, the addition of Pd greatly improves the overall stability of the nanocatalysts. These bimetallic formulations of CuPd/RGO can potentially be used in other applications such as fuel cells, catalytic oxidation of CO and other energy applications [245].

Bjelić et al. studied the activity, selectivity and reaction mechanisms of heterogeneous catalysts of noble and transition metals, on neutral supports (carbon) for the hydrodeoxygenation (HDO) of eugenol, selected as the model compound of the lignin blocks. The reactions proposed above for HDO phenols with Ru/C have also proved valid for other metals such as Pd, Pt and Rh and Cu or Ni. Ru/C catalysts showed the optimal HDO performances, followed by Rh/C, which above all showed outstanding hydrogenation (HYDR) activity. Instead, platinum and palladium displayed lower deoxygenation (de-OX), united with a moderate activity towards HYDR. The adsorption and desorption constants for H_2_, aromatics and saturated species were found, displaying a strong adsorption/dynamic influence on overall kinetics. The minimum HYDR/de-OX ratio was observed in the case of Ru/C catalyst, while the maximum in the case of Pd/C one. Furthermore, it was demonstrated that methoxy groups are more facile to detach from the aromatic rings, in comparison with the case of saturated compounds. Generally, the activation barrier for HYDR reactions is lower compared to those for de-OX ones. Hence, higher temperatures are necessary to achieve selective de-OX reactions, while higher pressures favor the HYDR reactions by increasing hydrogen coverage on the catalyst surface [246].

Since Fe is more abundant and cheaper than Cu and Ni, it is likely an alternative to noble metals in biomass conversions. Jiang Li et al. synthesized catalysts by simultaneous pyrolysis of graphite, carbon nitride and iron precursors on activated carbon, for the hydrodeoxygenation of biomass-derived 5-hydroxymethylfurfural to 2,5-dimethylfuran (DMF). The effect of pyrolysis temperature, support and iron content as well as reaction parameters such as H2 temperature and pressure were investigated. The highest yield of DMF was 85.7%, obtained at 240 °C for 3 h. The study of the reaction mechanism showed that 5-methylfurfural is the possible intermediate. The stability of the catalyst thus prepared could be improved following treatment with aqueous solution of HCl, which should selectively remove the inactive, agglomerated and unprotected iron particles generated during the pyrolysis process [247].

Gang Yuan et al. focused on the valorization of carboxylic acids derived from biomass for drop-in fuels with the contribution of renewable sources. In particular, Pt@Ir nanotorns were synthesized on 3D porous carbon fiber paper for anodes used for the electrocatalytic transformation of carboxylic acids into hydrocarbons usable in fuels. The core-shell anode is synthesized through electrodeposition of Pt nanotorns on carbon fiber paper followed by galvanic deposition of a thin layer of Ir nanotorns on the surface. Pt@Ir showed a significantly higher yield for hydrocarbon production than the reference commercial Pt/C catalyst. A self-supporting Pt@Ir NT catalyst with core-shell structure was also synthesized by controllable electrodeposition of Pt NT followed by the galvanic deposition of Ir on its surface in aqueous solutions, directly used as anode for the conversion of octanoate to hydrocarbons, in particular C7 hydrocarbons [248].

Lignin depolymerization is a pivotal process for the transformation of biomasses into smaller organic building blocks with high-added value. Guanqun Han et al. reported a photocatalytic system, constituted by ultrathin cadmium selenide nanosheets functionalized with metals of the first transition row (M/CdS), for the direct photocleavage of lignin-derived model compounds into smaller aromatics. M/CdS reducing power was used to simultaneously generate H_2_, thus eliminating the need for sacrificial reagents and contemporarily maximizing the energy conversion efficiency. It has also been shown that, by tuning the photocatalytic conditions, it is possible to increase catalyst selectivity towards diverse products, maintaining excellent selectivity. Several experiments were conducted to understand the mechanism of each catalytic process, highlighting the roles of either the solvent or the base in the photocleavage of the β-O-4 bond in lignin upgrade process. Lignin-derived model compounds photocatalytic oxidation and cleavage reactions were then reported to provide three categories of products, with the highest selectivity obtained with 2D cocatalyst/CdS ultra-thin nanosheets. Several cocatalysts (i.e., Mn, Fe, Co, Ni and Cu) deposited onto CdS showed outstanding performances for either alcohol oxidation or hydrogen evolution reactions under visible light irradiation. By modifying the reaction mixture, constituted by a solvent and a base, it is possible to enhance the selectivity towards 2-phenoxy-1-phenylethanone from the oxidation of 2-phenoxy-1-phenylethanol with an almost unitary yield in acetonitrile using Ni/CdS catalyst. The chemical oxidation of 2-phenoxy-1-phenylethanone could head to the generation of phenol and benzoic acid, instead. In contrast, photocatalysis conducted in CH_3_CN/H_2_O mixture, produced 2-phenoxy-1-phenylethanone along with the products from β-O-4 bond cleavage, acetophenone and phenol. Increasing the pH of the reaction mixture, i.e., by adding a 0.1 M KOH solution aliquot, could cause the complete photocleavage of 2-phenoxy-1-phenylethanol to acetophenone and phenol. One possible mechanism involves hydrogen species adsorbed on Ni/CdS during photocatalysis [249].

## 4. Conclusions

In the well explored field of research and development of new catalytic green routes towards fine-chemicals production, single-atom catalysts (SACs), in which all metal species are atomically dispersed on a solid support, and which often consist of well-defined mononuclear active sites, are expected to bridge homogeneous and heterogeneous catalysts for liquid-phase transformations of various substrates [250]. The relationship between catalytic performance and the role, if any, of the heterogeneous support, in terms of its valence state, coordination environment and anchoring chemistry accessible for the stabilization of single atoms or metal nanoclusters of the catalyst active sites, needs to be carefully evaluated. In recent years, several authors have opened the path for the deeper comprehension of the way in which the molecular-level interactions between supports and active species can head up to the development of programmable structure–activity relationships, by providing several strategies for specific tuning of the catalytic performances from a mechanistic perspective, for example, anchoring bio-inspired active single-sites onto various solid supports [251].

The availability of different analytical and instrumental techniques, has recently further allowed the optimization of a molecular approach toward heterogeneous catalysis allowing us to furnish possible answers to the numerous questions and problems raised in this area of science, such as: (i) the evaluation of the number of active sites (and/or phases); (ii) the possibility of understanding, even *in situ*, surface mechanisms and/or surface characterization, thus satisfying the real need for the comprehension of both, the molecular way and the structure/activity relationships of the catalytic system [252].

Different types of microscopies (scanning/transmission electron microscopy) and spectroscopies (electron energy loss, X-ray absorption, IR and Raman spectroscopy) have been used to provide new insights into the nature of the structure–property relationships that could occur in prototypical heterogeneous catalytic reactions, under *operando* conditions [253], which are closely aligned with computational modeling of these solid systems. This approach can be used to better understand the atomic structure and elemental composition of, for example, MO-based nanocatalysts, the physiochemical properties of the support and catalyst/support interfaces, then acquiring a deep knowledge of the surface chemistry arising during experiments. The surface-science approach on well-defined surfaces has reached such a level of understanding through which it is possible to see the molecules dissociating and recombining on a surface to give the products, that are the elementary steps of each catalytic process, thus allowing the design of catalytic systems capable of obtaining ever higher levels of selectivity in the reactions by them catalyzed.

## 5. Perspectives

In the transition period that we are facing, passing from a non-renewable-based to a renewable/circular-based economy, strong efforts from the catalysis community (and more in general by the global scientific community) in improving process efficiency with a greener approach, are mandatory. All these endeavors directed towards the valorization of biomass wastes for their conversion into either commodities or fine chemicals, thus substituting oil-base feedstocks with sensible environmental advantages, should open the way to a greener and more sustainable chemical industry for the future. Currently, the direction that research has taken is the right one, as testified by the enormous amount of literature that has been produced in the last 20 years on the valorization of biomass wastes and on wastes in general. Such a topic will have a huge technological and economic impact if brought to an industrial level. Unfortunately, up to this moment there is a broad gap that must be closed between the lab scale and the industrial one; in order to fill this hole, the time needed might be quite long if a global coordination among scientists, companies and governments is missing. In our opinion, regarding whether such a condition will be fulfilled in the near future, mankind could still have a long future ahead; contrarywise, the technological level that humanity has reached along the centuries will be hardly maintained, with evident problems in food production, energy provision and public health, which are at the basis of civilization survival. Luckily the time is not over yet; therefore, considering the world as a large community, we have to work harder and pushing together to reach the goals needed to build up a global economy that reduce as much as possible the use of non-renewable sources and gradually point towards feedstocks more and more based on recovered and renewable sources.

## Figures and Tables

**Table 1:** Summary of the different activated carbons sources.

Entry	Biomass Origin	Refs.
1	Vine wood	[76]
2	Pomegranate wood	[77]
3	Soybean hull	[78]
4	Plantain peel	[79]
5	Coconut shell	[53,58,69,79]
6	Tomato waste	[77]
7	Langsat empty fruit bunch	[80]
8	Papaya peel	[81]
9	Papaya seeds	[64]
10	Rice husk	[57,82]
11	Beech	[69]
12	Pine wood	[69]
13	Corn cob	[64]
14	Peanut hull	[56]
15	Lemon peel	[63]
16	Chitosan	[83]
17	Olive stone	[66]
18	Oil palm	[57]

## Data Availability

Not applicable.
